# Evaluation of the clinical efficacy of vaginal treatment options for persistent high-risk human papillomavirus infection after excisional treatment of cervical high-grade squamous intraepithelial lesions: a systematic review and Bayesian network meta-analysis

**DOI:** 10.1186/s12985-023-02001-6

**Published:** 2023-03-20

**Authors:** Yiqian Tang, Qing Tong, Ning Dai, Cai Xu

**Affiliations:** 1 Second Clinical College of Medicine, Beijing University of Chinese Medicine, Beijing, 100029 China; 2 Dongfang Hospital, Beijing University of Chinese Medicine, Beijing, 100078 China; 3 Beijing University of Chinese Medicine, Beijing, 100029 China

**Keywords:** HPV, Vaginal treatment, Conization, Cervix

## Abstract

**Background:**

To evaluate the clinical efficacy of different vaginal administration on cervical persistent high-risk human papillomavirus (HR-HPV) infection after excisional treatment for high-grade squamous intraepithelial lesions (HSIL).

**Methods:**

Six databases (PubMed, EmBase, Cochrane Central, China Knowledge Network database, China Biomedical Literature Service, and WanFang database) were searched to collect randomized controlled trials (RCTs) of various types of vaginal administration compared to no treatment on persistent HR-HPV infection after HSIL excisional treatment, and comprehensive analysis of the clearance of different drugs on HR-HPV was performed using Bayesian reticulation meta-analysis.

**Results:**

The study analyzed the efficacy of eight interventions, including Interferon, Baofukang, Paiteling, Bletilla striata Sanhuang Powder, Lactobacilli vaginal capsules, Fuanning + Interferon, Interferon + Lactobacilli vaginal capsules, and Interferon + Baofukang, on the clearance of HR-HPV after excisional treatment through pooling and analyzing data from 52 RCTs. The results of the study demonstrated that Interferon + Lactobacilli vaginal capsules [OR 16.0 (95% CIs 8.1–32.0)], Interferon + Fuanning [OR 16.0 (95% CIs 1.1–52.0)], and Interferon + Baofukang [OR 14.0 (95% CIs 6.8–28.0)] were all found to significantly improve postoperative HR-HPV clearance rates when compared to no treatment. Furthermore, when studies with high-risk bias were excluded, Interferon + Lactobacilli vaginal capsules [OR 8.6 (95% CIs 4.7–19.0)] and Interferon + Baofukang [OR 22.0 (95% CIs 8.7–59.0)] were still found to be positively associated with increased postoperative HR-HPV clearance rate. Additionally, the study´s results also indicate that Interferon + Baofukang was effective in enhancing the postoperative HR-HPV clearance rates, mainly when the studies were restricted to a follow-up period of at least 12 months [OR 9.6 (95% CIs 2.9–34.0)]. However, it is important to note that the majority of the trials (29 out of 52, 51.6%) were rated as moderate to high risk of bias, and the certainty of the evidence was moderate to very low.

**Conclusion:**

The application of various forms of vaginal administration, except for individual use of Lactobacilli vaginal capsules, is more efficacious than no treatment in patients with cervical persistent HR-HPV infection after excisional treatment. However, all of the estimates of the effect size for change in the efficiency of HR-HPV clearance are uncertain. Our confidence in effect estimates and ranking of treatments is low, which needs larger, more rigorous, and longer follow-up RCTs to resolve.

**Supplementary Information:**

The online version contains supplementary material available at 10.1186/s12985-023-02001-6.

## Background

The World Health Organization reports [1] that cervical cancer (CC) is the third most prevalent tumor in women worldwide and is one of the primary causes of tumor death in women in developing countries. CC develops gradually from the cervical squamous intraepithelial lesion (SIL). The primary etiology of SIL [2] is infection with Human Papillomavirus (HPV), particularly persistent infection with high-risk Human Papillomavirus (HR-HPV) strains, which instigates aberrant cellular proliferation and genomic instability. If left unchecked, these persistent HPV infections can progress to malignant transformation.

HPV is a small, envelope-free, double-stranded, ring-shaped DNA virus. Most HPV infections are transient and are often spontaneously cleared by the immune system within 24–48 months after first detection [3]. However, persistent infection with some high-risk HPVs can integrate into the host genome, leading to changes in gene structure and function and causing carcinogenesis [3, 4]. The HPV genome expresses early transcriptional programs encoding E1, E2, E4, E5, E6, and E7, which regulate the viral life cycle, and late transcriptional programs encoding proteins, major and minor capsid proteins L1 and L2, which are genome wrapping and viral particle assembly [5]. E6 and E7 are most important because they are oncoproteins that repress the tumor suppressors p53 and pRb, respectively, which disrupt the activation of the apoptotic pathway and promote cell proliferation [6]. During cancer development, viral integration into the host genome usually results in the loss of E2, E4, E5, L1, and L2 expression. In contrast, constitutive expression of E6 and E7 oncogenes is essential for cancer cell survival and growth [7].

HSIL is considered a precancerous lesion of the cervix, and if not treated appropriately, about one-third of the lesions may progress to invasive cervical cancer. The current treatment options for HSIL include surgical intervention, such as total hysterectomy and cervical excisional treatment including, cold knife conization (CKC) and loop electrosurgical excision procedure (LEEP), and non-surgical physical therapy, such as laser ablation, electrocoagulation, and cryotherapy. Excisional treatment is widely considered the preferred modality [1]. However, recent studies have highlighted the limitations of these excisional techniques in achieving complete resolution of persistent HPV infection, and the potential for long-term adverse effects resulting from persistent infection cannot be disregarded.

Song et al. [8] found that patients with persistent HR-HPV infection after LEEP had a higher risk of recurrence of HSIL. Fan et al. [9] also showed that some patients have persistent HPV infection or cytologic abnormalities after LEEP, a high-risk factor for HSIL recurrence and progression. A French retrospective study with a five-year follow-up period [10] also pointed out that the persistence of HPV after cervical conization was directly associated with an increased risk of recurrence in patients within 5 years. In the study by Nagai et al. [11], 5 of 11 (45.5%) patients with persistent positive HPV DNA after conization treatment developed cervical intraepithelial neoplasia (CIN) recurrence. At the same time, none of the 45 patients with persistent negative HPV DNA after conization recurred. In the study by Bruno et al. [12], 40% of CIN recurrences occurred in women who were margin-negative but persistently infected with HPV 16 after excisional treatment.

An extensive body of literature consisting of randomized controlled trials (RCTs) has been published on the postoperative pharmacological management of cervical excisional treatment. Various novel antiviral medications have been developed for this purpose. The majority of these studies have focused on the vaginal administration of these medications. Despite these efforts, no definitive therapy has been conclusively shown to effectively eradicate persistent HR-HPV infection in the cervix post-excisional treatment for HSIL, and the long-term adverse effects of these treatments remain uncertain. To date, no meta-analysis has been conducted to consolidate the findings from these RCTs.

In order to conduct a head-to-head study of this component, a systematic review and network meta-analysis of the clinical efficacy of vaginal administration in the management of persistent cervical HR-HPV infection following excisional treatment for HSIL was conducted utilizing an evidence-based scientific research approach. This research-based and methodical analysis will aid clinicians in gaining a greater understanding of the various treatment options currently available for HSIL patients with persistent HR-HPV infection.

## Methods

This systematic review with meta-analysis was conducted in accordance with the recommendations of PRISMA 2020 [13] and the PRISMA extension statement of the network meta-analysis (PRISMA-NMA) [14].

### Protocol and registration

The study protocol has been pre-registered with PROSPERO. (PROSPERO registration number: CRD42022287077). Deviations from the pre-registration are reported in the online supplementary material. (Available from: https://www.crd.york.ac.uk/prospero/display_record.php?ID=CRD42022287077).

### Study program

#### Study population

Patients who were diagnosed with HSIL via colposcopic examination prior to excisional treatment, or were diagnosed with HSIL via postoperative pathological analysis in conjunction with HR-HPV infection, were included in the study.

#### HSIL diagnostic criteria

According to the 2014 World Health Organization recommendations [15, 16]for the nomenclature of squamous intraepithelial lesions (SIL), a secondary classification was utilized to differentiate between LSIL and HSIL. LSIL comprises CIN I, CIN II p16 (-), while HSIL comprises CIN II p16 (+), and CIN III.

#### Study type

1. Inclusion criteria: Included in RCTs utilizing pharmacological interventions administered via vaginal administration following excisional treatment of the cervix, focusing on only utilizing results from clinical trials published in peer-reviewed journals
2. Exclusion criteria: Excluded studies for which a full-text version was not available or where data was missing, as well as any other systematic reviews that have been previously conducted.

#### Type of intervention

This study included RCTs that evaluated the efficacy of postoperative vaginal drug interventions as an excisional treatment for HSIL, either utilized alone or in combination with other drugs. Eligible trials included those utilizing any dosage of drug treatment, with no restrictions on the type or number of drug combination regimens employed. The drug or combination intervention in the trial had to be comparable to the control or placebo, and non-pharmacological studies were excluded. The control group in these trials included the use of a placebo, blank (no treatment) and the drugs themselves.

#### Outcome metrics

The primary outcome evaluated in this study was the drug treatment's efficacy on HR-HPV clearance as compared to a control group receiving blank (no treatment), other treatment groups, or a placebo group. The inclusion criteria for trial results included at least one of the following: HR-HPV clearance or viral load. Additionally, the follow-up period for each participant had to be a minimum of six months to be included in the study [17].

### Search strategy

Six databases, PubMed, EmBase, Cochrane Central, China Knowledge Network database (CNKI), China Biomedical Literature Service (SinoMed), and WanFang database (WanFang) were searched by computer. The search date was from the establishment of the database to February 2022. Search strategies for all databases are shown in the Additional file 1. There are no language restrictions.

### Research selection process

The search results were imported into the software program Note Express (version: 3.5.0.9054), and subsequently, all potentially pertinent articles were gathered and thoroughly evaluated. Two authors, who independently removed duplicates, reviewed the titles and abstracts of the database search results. Two investigators independently evaluated the abstracts discovered through the initial search by predefined criteria. Subsequently, two reviewers independently reviewed and assessed the full text of the articles in duplicate, utilizing the Cochrane Intervention System Evaluation Manual to determine which RCTs should be included in the study. Furthermore, discussions with independent expert professors helped the scholars reach a consensus.

### Data extraction

The following data were extracted from the included studies: (1) identifying information, including the first author’s name and the year of publication; (2) general information, including the background of the study, the sample size, and the duration of the study; (3) characteristics of the participants, including their excisional treatment preoperative cervical intraepithelial neoplasia status, age, and gender; (4) details of the interventions, including the type of drug, the specific drug name, the dosage, the frequency of administration, and the duration of treatment; and (5) outcomes of the interventions. Only trials that provided extractable data were included, and no additional information was requested from the authors.

### Data analysis

Data extraction was conducted independently by two researchers, who compiled the results into a Microsoft Excel spreadsheet, categorizing them as either positive or negative. The HPV viral load was determined using the Hybridized Capture II gene hybridization signal amplification system, with the ratio of relative light units (RLU) to cutoff (CO) as the indicator of viral load. A value of RLU/CO < 1.0 was classified as a negative result, while a value of RLU/CO ≥ 1.0 was considered a positive result. Due to the different settings of follow-up nodes in numerous reports, we selected the outcome indicator with the longest recorded follow-up time from the available reports to define efficacy.

### Quality assessment and risk of bias

This study was conducted by two researchers who worked independently to evaluate the quality of the assessment. Any discrepancies that were encountered during the process were resolved through a discussion. The Cochrane Handbook (RoB) [18] was utilized to evaluate the potential for bias by recording the procedures used to generate the randomization scheme and conceal allocation, as well as the implementation of blinded methods, blinded implementers, and results assessment. Furthermore, an examination of incomplete outcome data and any evidence of selective reporting of results was also conducted. Sample sizes that exceeded 100 were considered to be large sample studies [19]. Additionally, we assessed the certainty of evidence contributing to network estimates of the primary outcomes with the Grading of Recommendations Assessment, Development, and Evaluation (GRADE) framework [20].

### Data synthesis and statistical analysis

To explore the indirect treatment comparisons of the efficacy of each drug, we chose to utilize a Bayesian multiple treatment network meta-analyses with random effects. This approach has been found to offer several advantages when compared to conventional meta-analyses. Specifically, network meta-analyses often yield more accurate estimates, and enable the ranking of treatments in a way that facilitates clinical decision-making [21].

To thoroughly analyze the symmetry and geometry of the evidence on vaginal administration-related treatment regimens, we employed a network plot in which the nodes represented the number of study participants and the connection sizes. We used Stata 16.0 to generate a comparison-adjusted funnel plot to investigate the presence of publication bias among all available comparisons with either placebo or no treatment. To summarize the effect of each comparison, we calculated an odds ratio (OR) with 95% credible intervals (CIs), using a random effects model as a conservative estimate. We employed consistency models to investigate the correlation between direct and indirect evidence. To provide a treatment hierarchy, we utilized the treatment surface under the cumulative ranking curve (SUCRA) with a rank plot, considering the position and variance of all relative treatment effects. The SUCRA value was 0 when the treatment was the worst and 1 was the best [22]. To account for the standard deviations between studies, we modeled them using a uniform distribution between the 0 and 5 intervals. Furthermore, we employed Markov chain Monte Carlo methods, based on Gibbs sampling at 200,000 iterations per 4 chains, to calculate random effects models. We verified homogeneity and consistency using the node splitting method and the Bland–Altman method [23, 24]. We ranked treatments based on their relative effect corresponding to an arbitrary baseline for each iteration. Results were considered to differ when 95% of CIs did not include 1.0. Finally, we constructed a frequency table from these rankings and normalized it by giving the number of iterations of ranked probabilities, using standard diagnostics to assess convergence [25].

To evaluate the impact of various forms of prior knowledge on the outcomes, we conducted a sensitivity analysis. Initially, we considered only those studies with a low bias risk. Secondly, we performed subgroup analyses based on the duration of follow-up, specifically 6, 12, and 24 months, as HPV infection after excisional treatment typically clears within 3–6 months via autoimmunity, and recurrence tends to occur within 2 years [26, 27], which more accurately reflects the practical usage of the drug in “real-world” scenarios. Finally, we repeated our preliminary analyses using Bayesian models to examine further our obtained results' robustness. All analyses were conducted using R-evolution (version 4.2.0) and the gemtc package (version 1.0-1), as well as the rjags package (version 4-13), interfaced with OpenBUGS (version 3.2.3) for computational Markov chain Monte Carlo simulations.

## Results

**Fig. 1:**
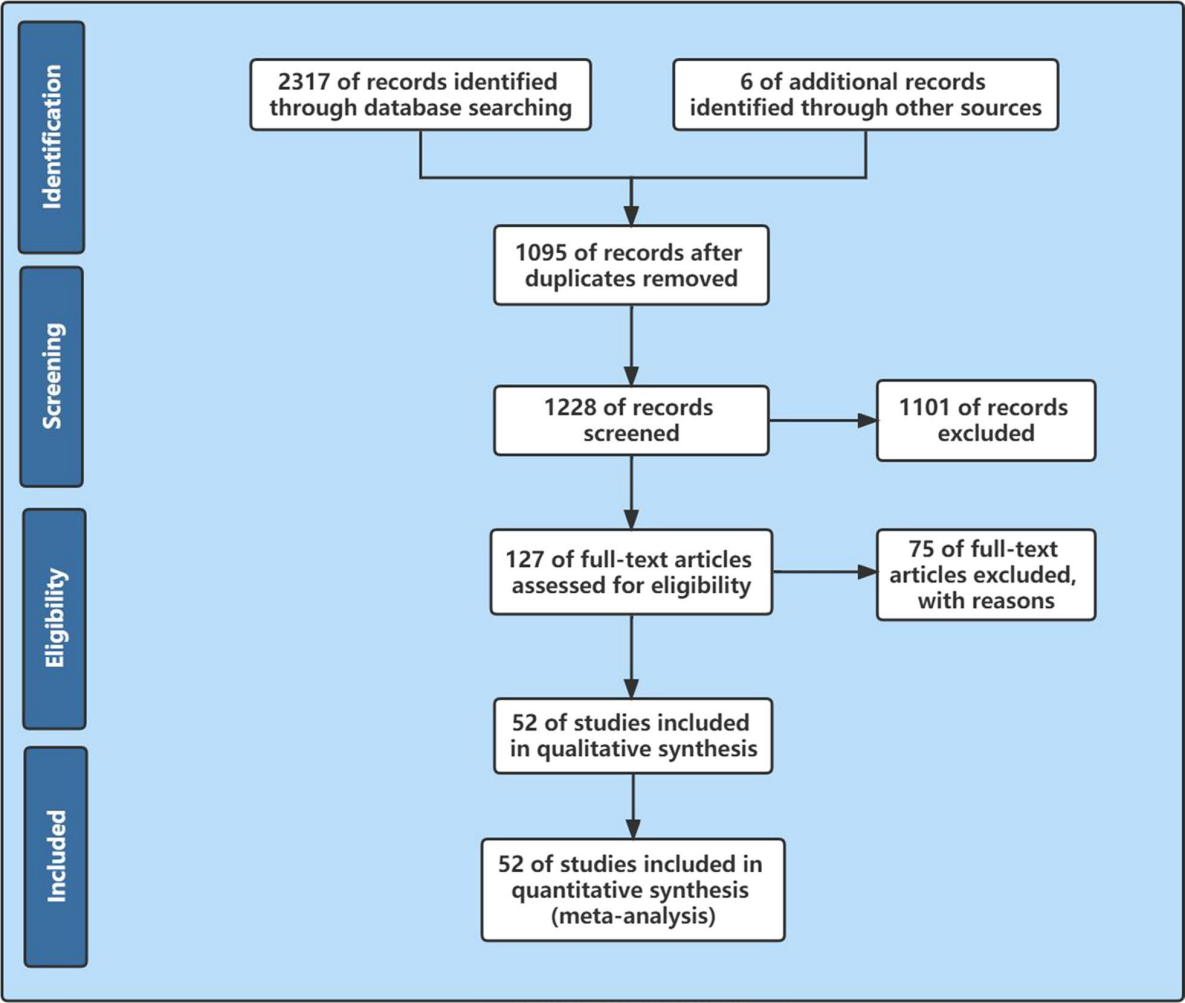
PRISMA Flow Chart

Of the 2323 records identified as a result of the search, 1095 duplicate publications were identified and removed. The abstract review team screened 1228 abstracts and selected 127 articles for full-text evaluation. Additional relevant articles were searched in the reference section of all included articles. A total of 52 articles were ultimately included in this study. Full-text articles not published in English were translated and evaluated according to the same criteria as the English articles. The process was summarized using the PRISMA flowchart (Fig. 1).

A total of 52 eligible studies, consisting of 52 independent randomized controlled trials, were identified, including 5460 patients. The characteristics of the 52 included trials are summarized in Table 1. The most recent study was published in 2022, with the earliest study beginning in 2011. The demographic and clinical traits of the included patients are typical of those with HSIL and HR-HPV infection, primarily consisting of women of childbearing age, with a mean age predominantly ranging between 21 and 67 years. Among the 52 included trials, 33 evaluated the effects of Interferon alone, 9 evaluated the effects of Baofukang alone, 1 evaluated the effects of Paiteling alone, 1 evaluated the effects of Bletilla striata Sanhuang Powder alone, 1 evaluated the effects of Lactobacilli vaginal capsules alone, 1 evaluated the effects of Fuanning + Interferon in comparison with Interferon, 1 evaluated the effects of Paiteling in comparison with Interferon, 2 evaluated the effects of Interferon + Lactobacilli vaginal capsules in comparison with Interferon, 2 evaluated the effects of Interferon + Baofukang in comparison with Baofukang, and 1 three-armed experiment investigated the effects of Interferon + Lactobacilli vaginal capsules versus Interferon + Baofukang versus Interferon.

**Table 1:** Characteristics of Trials Included in the Analysis and Summary Trial Quality Assessment

Source	No of patients	Age of treatment group (Y)	Age of control group (Y)	Cochrane collaboration risk of bias	Follow-up(month)	Type of intervention and dose
Yu2021	60	38.76 ± 5.27	37.48 ± 4.92	Low; unclear; low; low; low; low	6	Bletilla striata Sanhuang Powder (Bletilla striata, Coptis chinensis, Cortex phellodendri, Scutellaria baicalensis), 3 g per application, once every other day, for 2 months
Guan2017	80	39.61 ± 2.09	39.73 ± 2.16	Unclear; unclear; low; low; low; low	12	Baofukang, 1 capsule/d for 2 months
Meng2015	428	36.92 ± 2.9	37.22 ± 2.2	Low; unclear; low; low; low; low	24	Baofukang, 1 capsule/d for 2 months
Li2015	70	36.7 ± 4.5	37.1 ± 4.8	Low; unclear; low; low; low; low	6	Interferon, 1 pill/night for 3 months
WangXL2015	112	53.67 ± 9.32	54.66 ± 9.82	Low; unclear; low; low; low; low	12	Interferon (500,000 IU/pc), once every other day for 3 months
Feng2019	86	38.19 ± 6.54	38.32 ± 6.43	Low; unclear; low; low; low; low	6	Interferon, once/night, for 3 months
Cao2011	83	37.0 ± 3.7	38.0 ± 2.4	Unclear; unclear; low; low; low; low	12	Interferon, once every other day for 3 months
Jia2019	120	38.12 ± 4.10	37.29 ± 4.28	Low; unclear; low; low; low; low	6	Lactobacillus vaginal capsule, 1 capsule/night, 7d for 1 course of treatment, 4 courses of treatment in total
Han2014	96	Not mentioned	Not mentioned	Unclear; unclear; low; low; low; low	6	Interferon (800,000U/capsule), 1 capsule per night for 10d for 3 menstrual cycles
Cui2013	148	31.67 ± 6.04	32.97 ± 5.57	Unclear; unclear; low; low; low; low	12	Baofukang 1/per night for 3 months
WangX2015	60	41.3 ± 3.64	40.1 ± 3.51	Unclear; unclear; low; low; low; low	6	Interferon, 1 capsule/d every 2d for 3 months
Yu2022	94	44.3 ± 6.2	43.9 ± 5.9	Low; unclear; low; low; low; low	12	Interferon, 1 capsule every 2d for 3 months
Lv2020	82	37.86 ± 2.43	38.13 ± 2.87	Low; unclear; low; low; low; low	6	Baofukang, 1 capsule/d for 2 months
Cheng2017	96	45.27 ± 9.13	44.89 ± 10.21	Unclear; unclear; low; low; low; low	12	Interferon, 1 g every other day for 3 months
Zhong2019	92	40.62 ± 9.23	40.37 ± 8.10	Low; unclear; low; low; low; low	6	Baofukang, 1 capsule/d for 14 days
Zhao2021	55	36.52 ± 2.18	36.19 ± 2.17	Low; unclear; low; low; low; low	6	Interferon, 1 capsule once every other day for 2 months
Zhan2018	126	47.1 ± 3.6	46.3 ± 3.1	Unclear; unclear; low; low; low; low	12	Interferon, 1 capsule/dose, 1 time/2 days
Yu2017	78	43.24 ± 6.17	42.69 ± 7.12	Unclear; unclear; low; low; low; low	12	Interferon (100,000 IU/capsule), 1 capsule each time, every other day
Cui2020	180	40.99 ± 13.72	41.53 ± 13.81	Low; unclear; low; low; low; low	6	Interferon (800,000 U/capsule) 1 capsule each time/night for 3 months
Zhen2020	128	36.91 ± 3.22	36.84 ± 3.15	Low; unclear; low; low; low; low	6	Interferon, 1 capsule/d, every other day, for 2 months
Zhang2013	113	41.2 ± 5.6	42.4 ± 4.1	Unclear; unclear; low; low; low; low	12	Interferon (500,000 u/spig), 1 time/2d, 9 times as a course of treatment, 3 courses in total
Xu2014	82	42.83 ± 5.42	40.65 ± 5.39	Low; unclear; low; low; low; low	12	Interferon, 1 capsule/d, 2 times/d, 10 times/course, 3 courses of continuous treatment
Jiang2016	110	41.2 ± 4.7	40.7 ± 4.4	Low; unclear; low; low; low; low	6	Interferon, 1 capsule every other day for 3 months, discontinue during menstruation
Wu2021	164	37.10 ± 7.69	36.98 ± 7.23	Unclear; unclear; low; low; low; low	6	Interferon, 1 capsule/d every other day for 3 months
Gu2013	90	39.3	40.5	Unclear; unclear; low; low; low; low	12	Interferon, 1 capsule once a night for 1 month for 10d (stop using during menstruation) for 3 months
Liu2019	78	44.05 ± 7.12	43.26 ± 6.89	Low; unclear; low; low; low; low	12	Interferon (5 g/stick), 1 g/2d, continue treatment for 3 months
Wu2020	80	42.26 ± 7.89	43.78 ± 7.29	Low; unclear; low; low; low; low	12	Interferon, 1 g once every 2 days for 4 months
Li2014	80	34.6 ± 6.3	34.6 ± 6.3	Unclear; unclear; low; low; low; low	6	Interferon, 1 capsule/2d for 20d/M for 3 months
Zhu2014	144	32.5	32.5	Unclear; unclear; low; low; low; low	6	Interferon, 1 capsule every other day for 3 months
LiRH2015	41	24–65	24–65	Unclear; unclear; low; low; low; low	6	Interferon (0.5 g/stick), 0.1 g/d for 14d
Ye2017	80	35.42 ± 6.11	35.46 ± 6.03	Low; unclear; low; low; low; low	6	Interferon (100,000 IU/each), 2 capsules/d for 2 months
Liu2020	84	35.22 ± 3.34	35.08 ± 3.17	Low; unclear; low; low; low; low	6	Interferon, 1 capsule every other day, for 3 weeks
Pan2017	120	44.8 ± 5.8	44.3 ± 5.4	Low; unclear; low; low; low; low	6	Baofukang, 2 capsules/d, for a course of 16d, two courses in total
Chang2019	100	41.51 ± 5.31	42.32 ± 5.72	Low; unclear; low; low; low; low	6	Interferon (800,000 U/capsule), 1 capsule/d, for a course of 10d, two courses in total
He2017	116	36.79 ± 3.12	37.46 ± 3.39	Low; unclear; low; low; low; low	6	Interferon (100,000 IU/capsule), 1 capsule every other day for 2 months
Zhou2012	42	36.7 ± 3.2	36.7 ± 3.2	Unclear; unclear; low; low; low; low	6	Baofukang, 1 capsule/d for 15d
Wang2022	126	33.7 ± 9.0	33.6 ± 8.9	Low; unclear; low; low; low; low	6	Interferon, 4 times/d, for 3 months
Ji2021	100	33.82 ± 2.31	33.65 + 2.28	Low; unclear; low; low; low; low	6	Interferon, every other day for 3 months
Wang2010	148	36.5	35.7	Unclear; unclear; low; low; low; low	12	Baofukang, 1 per night, for 7 days
Ding2015	128	62.38 ± 8.11	63.51 ± 8.65	Low; unclear; low; low; low; low	6	Interferon (500,000 IU/pc), 1 pc every other day for 1 month
Yao2018	80	36.4 ± 3.8	35.6 ± 5.4	Unclear; unclear; low; low; low; low	6	Paiteling, once every other day, 10 times for one course of treatment, 3 courses in total
Cao2016	60	32.6 ± 5.4	32.6 ± 5.4	Unclear; unclear; low; low; low; low	6	Baofukang, 1 capsule once/day for 1 month
Zhao2017	96	32.7 ± 4.3	33.2 ± 5.4	Unclear; unclear; low; low; low; low	12	Interferon (800,000 IU/capsule)for 3 months
Hao2016	113	28–50	28–50	Unclear; unclear; low; low; low; high	6	Interferon, 1 capsule/d, 10d 1 course, 3 courses in total
Su2021	90	37.23 ± 12.60	37.22 ± 12.58	Unclear; unclear; low; low; low; low	6	Interferon (800,000 IU/capsule), 10d 1 course, 3 courses in total
WangCL2015	100	20–60	20–60	Unclear; unclear; low; low; low; low	12	Intervention (100,000 IU/capsule) + FUANNING (the formula consists of 15 g of Radix Sophorae Favescentis, 15 g of Phellodendron Chinense, 15 g of Fructus Cnidii, 10 g of Cyrtomium Fortunei, 10 g of Radix Clematis, 10 g of Rhizoma, 30 g of fructus Carpesii Abrotanoidis, 3 g of Peppermint, 3 g of Borneol), once every other day for 3 months Interferon (100,000 IU/capsule), 1 capsule/d, once every other day for 3 months
Peng2021	90	39.61 ± 4.28	39.24 ± 4.09	Unclear; unclear; low; low; low; low	6	1. Interferon (100,000 IU), 1 capsule/d, once every other day for 3 months 2. Lactobacilli vaginal capsules (0.25 g), 1 capsule/d + Interferon (100,000 IU), 1 capsule/d, once every other day for 3 months 3. Interferon (100,000 IU) + Baofukang, 1 capsule/d for 3 months
Zhou2021	60	40.18 ± 5.13	40.26 ± 5.01	Low; unclear; low; low; low; low	6	1. Lactobacilli vaginal capsules (0.25 g/capsule) + Interferon (100,000 IU), 1 capsule/dose, 1 time every 2 days, 10 capsules for 1 course of treatment, total 3 courses of treatment 2. Lactobacillus vaginal capsules (0.25 g/capsule), 1 capsule/dose, 1 time every 2 days, 10 capsules for 1 course of treatment, total 3 courses of treatment
Chen2017	100	43.17 ± 6.93	44.05 ± 6.95	Low; unclear; low; low; low; low	24	1. Baofukang, 2 capsules/d for 16d per month for 3 months 2. Baofukang, 2 capsules/d for 16d per month + Interferon (100,000 IU), 1/d, for 3 months
Zhong2021	187	45.56 ± 5.15	46.21 ± 4.76	Low; unclear; low; low; low; low	12	1. Baofukang, 2 capsules/d for 3 months 2. Baofukang, 2 capsules/d + Interferon (10 g/pc), 1 g once every other day, 6 to 10 times as a course of treatment, 3 months in total
Huang2017	84	38.2 ± 4.1	37.9 ± 4.2	Low; unclear; low; low; low; low	6	1. Lactobacilli vaginal capsules (0.25 g/capsule), 1 capsules/d + Interferon (100,000 IU), 1 capsule once every other day for 3 months 2. Interferon (100,000 IU), 1 capsule once every other day for 3 months
Zhao2019	100	31.76 ± 3.47	32.17 ± 3.16	Unclear; unclear; low; low; low; low	12	1. Interferon, 1 capsule once every other day for 6 weeks 2. Paiteling, with continuous medication for 3 d and discontinued for 4 d for 6 weeks

**Fig. 2:**
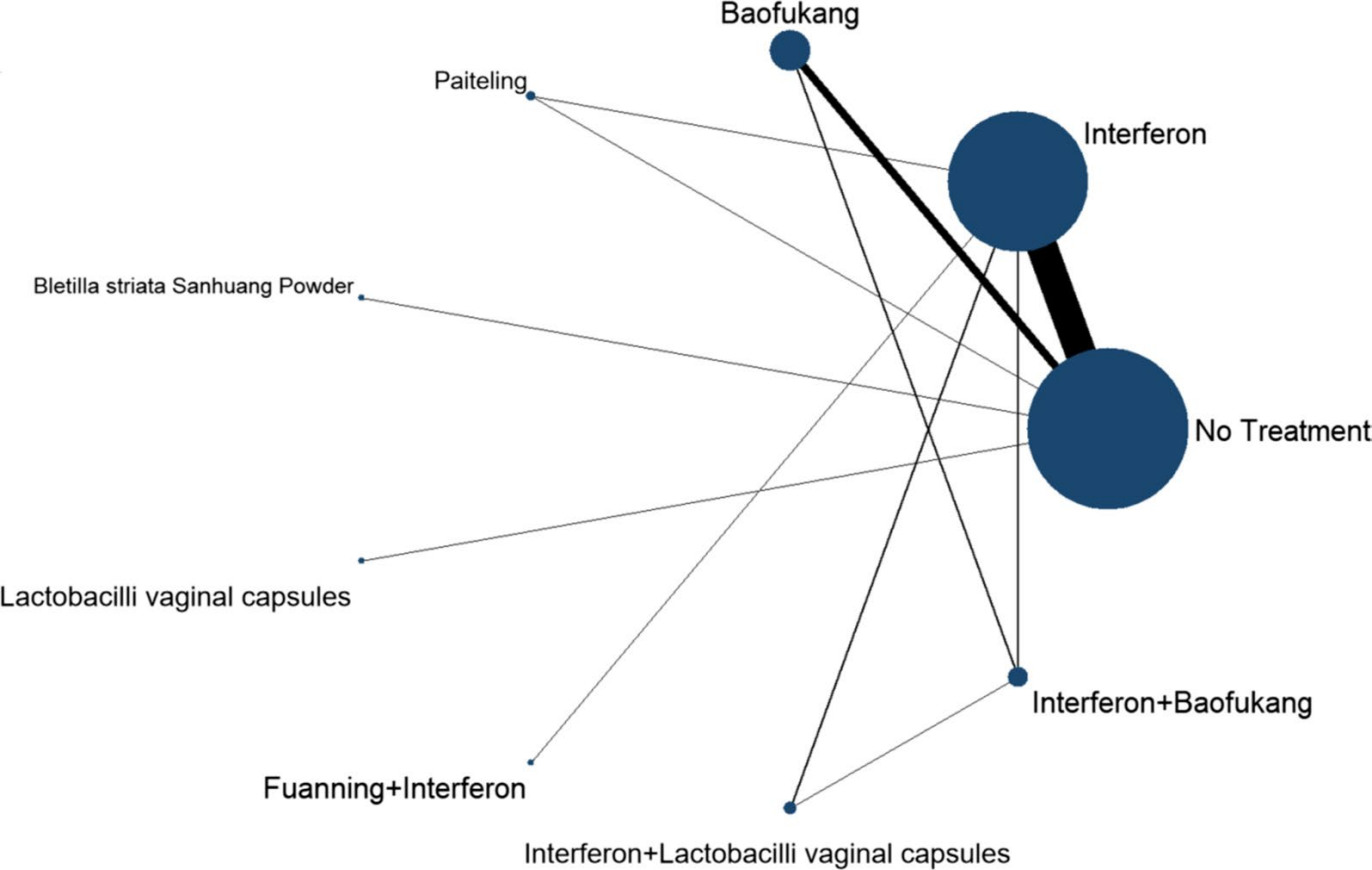
Network Plot of Interventions. Each circle corresponds to a scenario included in the analysis, and its area is proportional to the statistical information (cumulative number of events). Each line represents a direct comparison between interventions, and the thickness corresponds to the number of available direct intra-trial comparisons

Notably, there was only 1 large trial (1.92%) among the included literature. All trials employed random sequence generation, while only 23 trials specified the random assignment method, 29 trials did not mention it. None of the trials were blinded, but bias is unlikely for the primary outcome indicator, HR-HPV clearance, as viral clearance is an endpoint that is not susceptible to patient, physician, or outcome assessor bias. The trial duration varied from 1 to 3 months, with a follow-up period ranging from 6 to 24 months after excisional treatment. The Cochran Collaboration tool was used to assess the risk of bias, and it was found that 29 trials (51.6%) had a moderate to high risk of bias. Given the comparability of study design, outcome measures, patient population, and inclusion and exclusion criteria, a quantitative synthesis of the evidence by network meta-analysis was deemed appropriate. The assumption of homogeneity and consistency was confirmed (Fig. 2).

### GRADE of certainty of evidence

We incorporated the GRADE judgments in Table 2. The level of certainty regarding the relative treatment effects of efficacy and acceptability varied; it was moderate to low for the comparisons involving Interferon, Baofukang, Lactobacilli vaginal capsules, and Paiteling, and low to very low for comparisons involving Bletilla striata Sanhuang Powder, Fuanning + Interferon, Interferon + Baofukang, and Interferon + Lactobacilli vaginal capsules.

**Table 2:** Summary of confidence in effect estimates and ranking of treatments

Comparison	Nature of the evidence	Confidence rating	Reason(s) for downgrading
IFN:NT	Mixed	Moderate	Study limitations^a^
IFN:BFK	Indirect	Low	Study limitations^a^, Imprecision^b^
IFN:BSP	Indirect	Low	Study limitations^a^, Imprecision^b^
IFN:LVC	Indirect	Low	Study limitations^a^, Imprecision^b^
IFN:PTL	Mixed	Low	Study limitations^a^, Inconstantly^c^
IFN:FANIFN	Mixed	Moderate	Study limitations^a^
IFN:IFNBFK	Mixed	Moderate	Study limitations^a^
IFN:IFNLVC	Mixed	Moderate	Study limitations^a^
BFK:NT	Mixed	Moderate	Study limitations^a^
BFK:BSP	Indirect	Low	Study limitations^a^, Imprecision^b^
BFK:LVC	Indirect	Low	Study limitations^a^, Imprecision^b^
BFK:PTL	Indirect	Moderate	Study limitations^a^
BFK:FANIFN	Indirect	Moderate	Study limitations^a^
BFK:IFNBFK	Mixed	Moderate	Study limitations^a^
BFK:IFNLVC	Indirect	Moderate	Study limitations^a^
BSP:NT	Mixed	Very low	Study limitations^a^, Imprecision^b^, Inconstantly^c^
BSP:LVC	Indirect	Low	Study limitations^a^, Imprecision
BSP:PTL	Indirect	Low	Study limitations^a^, Imprecision^b^
BSP:FANIFN	Indirect	Low	Study limitations^a^, Imprecision^b^
BSP:IFNBFK	Indirect	Low	Study limitations^a^, Imprecision^b^
BSP:IFNLVC	Indirect	Low	Study limitations^a^, Imprecision^b^
LVC:NT	Mixed	Low	Study limitations^a^, Imprecision^b^
LVC:PTL	Indirect	Moderate	Study limitations^a^
LVC:FANIFN	Indirect	Moderate	Study limitations^a^
LVC:IFNBFK	Indirect	Moderate	Study limitations^a^
LVC:IFNLVC	Indirect	Moderate	Study limitations^a^
PTL:NT	Mixed	Moderate	Study limitations^a^
PTL:FANIFN	Indirect	Low	Study limitations^a^, Imprecision^b^
PTL:IFNBFK	Indirect	Low	Study limitations^a^, Imprecision^b^
PTL:IFNLVC	Indirect	Low	Study limitations^a^, Imprecision^b^
FANIFN:NT	Indirect	Moderate	Study limitations^a^
FANIFN:IFNBFK	Indirect	Low	Study limitations^a^, Imprecision^b^
FANIFN:IFNLVC	Indirect	Low	Study limitations^a^, Imprecision^b^
IFNBFK:NT	Indirect	Moderate	Study limitations^a^
IFNBFK:IFNLVC	Mixed	Very low	Study limitations^a^, Imprecision^b^, Inconstantly^c^
IFNLVC:NT	Indirect	Moderate	Study limitations^a^
Ranking of treatments		Low	Study limitations^d^, Imprecision^e^

*IFN* Interferon, *BFK* Baofukang, *PTL* Paiteling, *BSP* Bletilla striata Sanhuang Powder, *LVC* Lactobacilli vaginal capsules, *FAN* + *IFN* Fuanning + Interferon, *IFN* + *LVC* Interferon + Lactobacilli vaginal capsules, *IFN* + *BFK* Interferon + Baofukang, *NT* No treatment

^a^Dominated by evidence at high or moderate risk of bias

^b^Confidence intervals include values favoring either treatment

^c^Predictive intervals for treatment effect include effects that would have different interpretations (there is additionally no convincing evidence for the plausibility of the transitivity assumption)

^d^51.6% of the information is from studies at moderate or high risk of bias

^e^Moderate level of imprecision in the analysis

None of the effect estimates was accompanied by high confidence, 13 had moderate confidence, 17 had low confidence, and 2 had very low confidence. Our confidence in ranking the 8 interventions is low, due to downgrading for study limitations and imprecision. The detailed assessment and reasons for downgrading for each item are shown in Additional file 3.

### Primary outcome

**Fig. 3:**
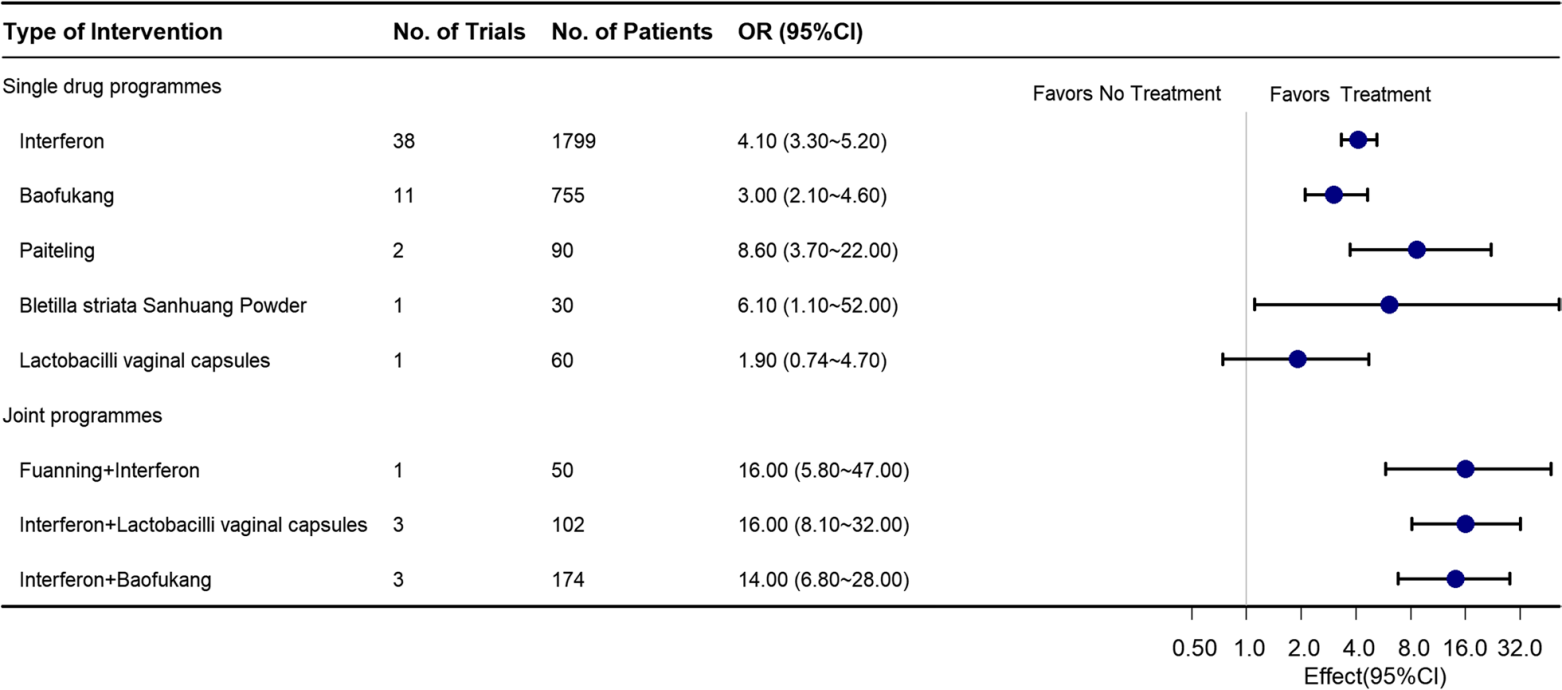
Forest Plot for the Estimates of Clinical Efficacy of Interventions. The figure depicts the ORs and 95% CIs for the comparison between the interventions and NT (No Treatment)

The study comparison network diagram, as depicted in Fig. 3, presents a comprehensive representation of the comparisons of the various interventions analyzed within the network. The trial interventions that were most commonly investigated include Interferon, Baofukang, among others, for a total of 8 interventions. It is evident that most interventions only have indirect comparisons and lack direct comparative evidence.

As depicted in the forest plot (Fig. 3), the estimated treatment effects of the eight interventions on HR-HPV clearance rates following excisional treatment are presented, with low overall statistical heterogeneity (I^2^ = 8%). Furthermore, a statistical examination of the results shows significant differences in the effects between the interventions and the control group, which received no treatment (NT). However, an exception to this trend is observed in the case of Lactobacilli vaginal capsules, whose confidence interval includes a value of 1 compared to the control group.

**Fig. 4:**
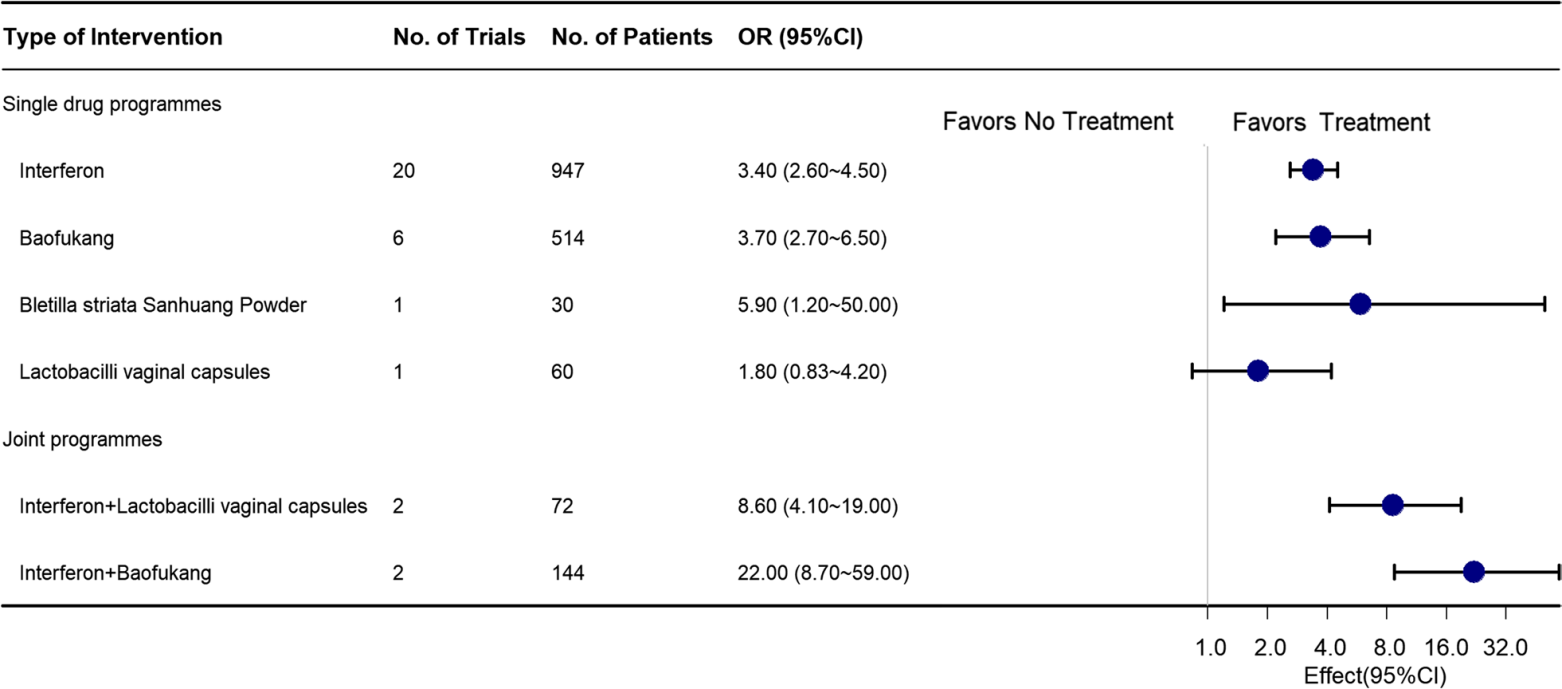
Forest Plot for Clinical Efficacy of Interventions That Excluded Trials at High Risk of Bias. The figure depicts the ORs and 95% CIs for each intervention compared to no treatment that excluded trials at high risk of bias

**Fig. 5:**
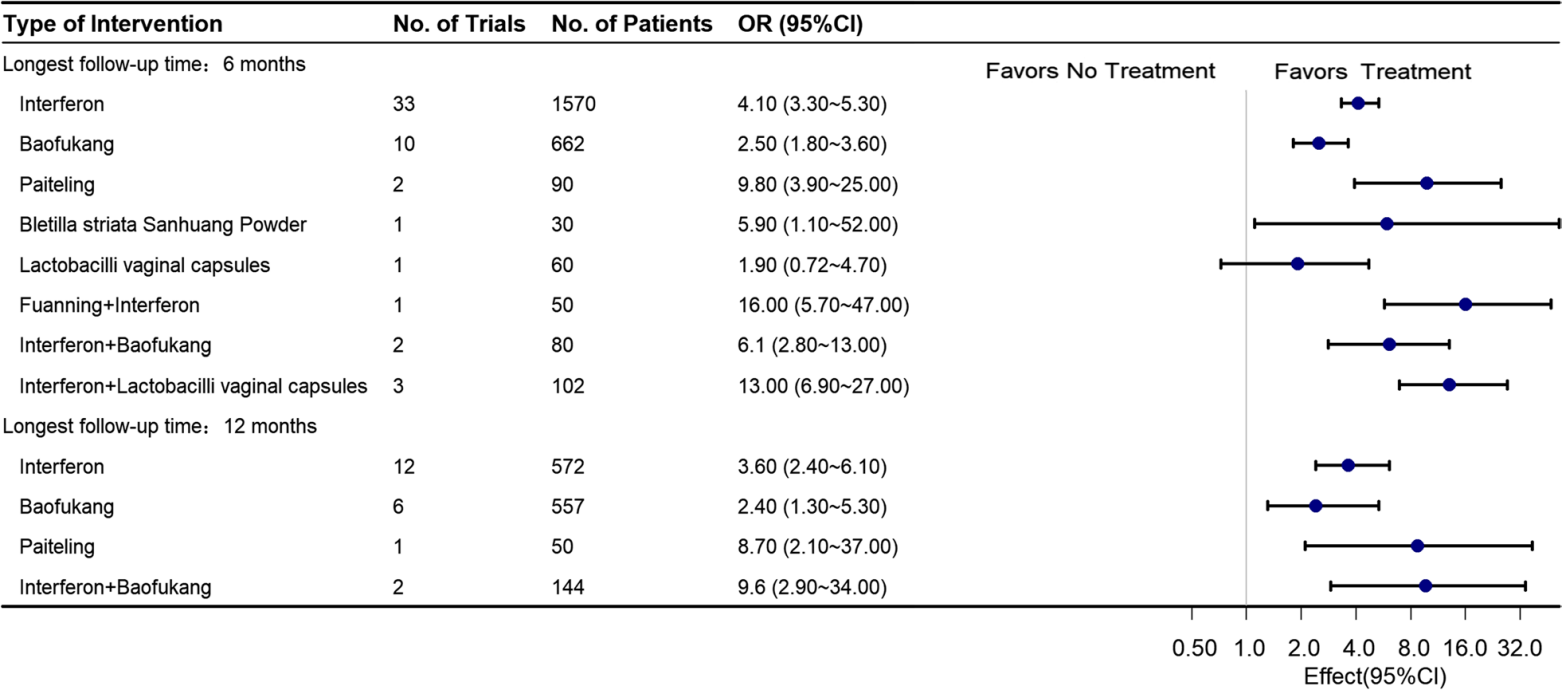
Forest Plot for the Estimates of Clinical Efficacy of Interventions for Subgroups of Follow-up. The figure depicts the ORs and 95% CIs for each intervention compared with no treatment when subgroup analysis was performed, depending on the longest follow-up time

A comparison of postoperative HR-HPV clearance rates with the efficacy of the control group (NT) reveals that the interventions of Interferon + Lactobacilli vaginal capsules [OR 16.0 (95% CIs 8.1–32.0)], Interferon + Fuanning [OR 16.0 (95% CIs 1.1–52.0)], and Interferon + Baofukang [OR 14.0 (95% CIs 6.8–28.0)] all significantly improve postoperative HR-HPV clearance rates. Additionally, we conducted a series of prespecified subgroups. Upon exclusion of studies with high-risk bias, 28 randomized controlled trials were included, enrolling a total of 2131 patients treated and yielding low overall statistical heterogeneity (I^2^ = 0%) (as illustrated in the network plot in Additional file 2: Fig. S1-1). Notably, Interferon + Fuanning was not included in the analysis, but Interferon + Lactobacilli vaginal capsules [OR 8.6 (95% CIs 4.7–19.0)] and Interferon + Baofukang [OR 22.0 (95% CIs 8.7–59.0)] were still found to be associated with improved postoperative HR-HPV rates levels (Fig. 4). Furthermore, when limiting studies to studies with follow-up periods of at least 12 months, 18 randomized controlled trials were included, enrolling 1399 patients (network plot appears in Additional file 2: Fig. S1-2). Interferon + Baofukang remained significantly associated with improved postoperative HR-HPV clearance rates [2 RCTs, OR 9.6 (95% CIs 2.9–34.0)], with low overall statistical heterogeneity (I^2^ = 19%) (Fig. 5).

**Fig. 6:**
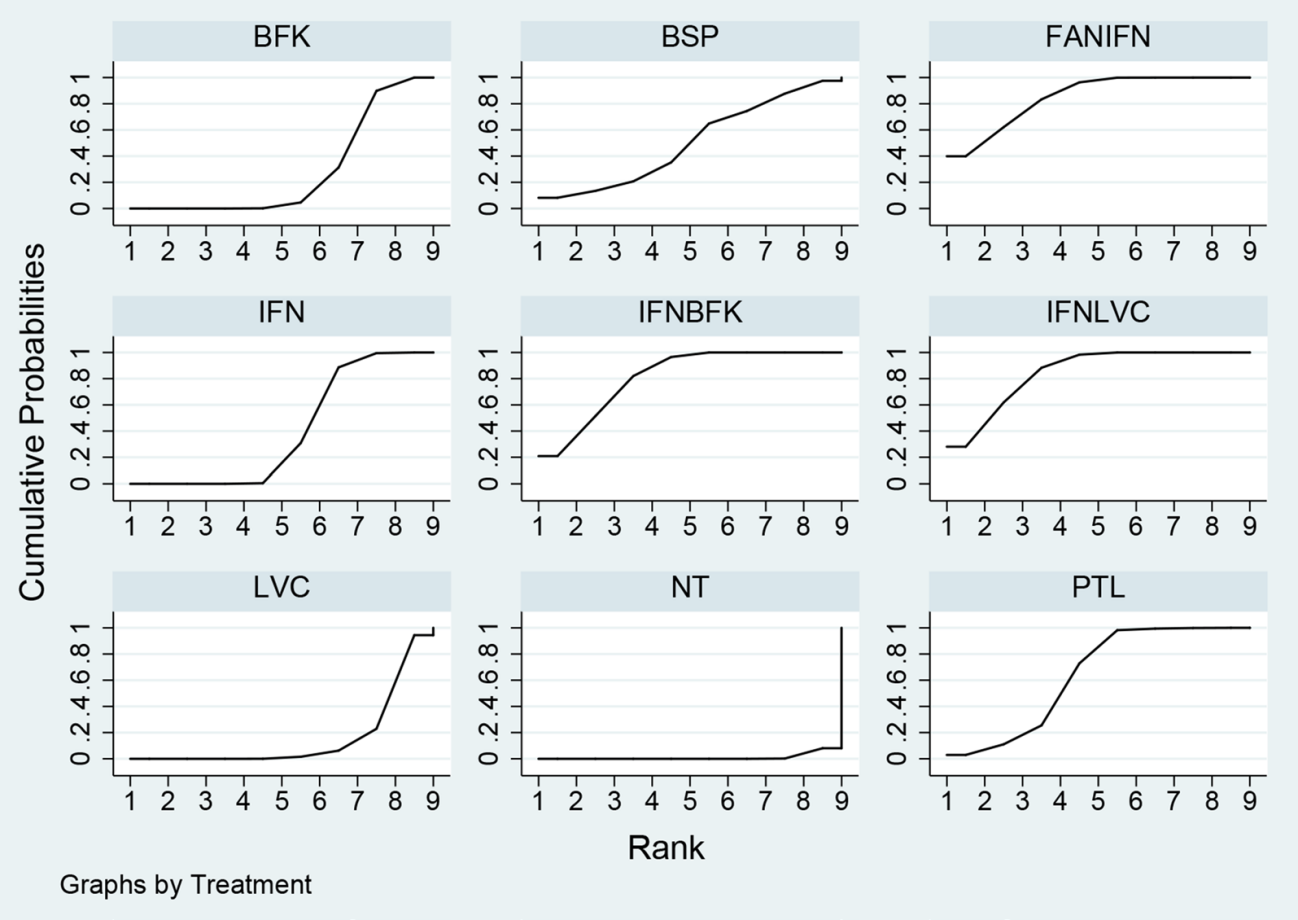
Surface under the Cumulative Ranking Curve. The cumulative ranked area under the curve gives the probability that a treatment will be most effective for HR-HPV clearance after HSIL excisional treatment. The larger the surface under the curve, the higher the probability of achieving and ranking. *IFN* Interferon, *BFK* Baofukang, *PTL* Paiteling, *BSP* Bletilla striata Sanhuang Powder, *LVC* Lactobacilli vaginal capsules, *FAN* + *IFN* Fuanning + Interferon, *IFN* + *LVC* Interferon + Lactobacilli vaginal capsules, *IFN* + *BFK* Interferon + Baofukang, *NT* No treatment. Rank Probability (preferred direction = 1)

Upon analyzing all available trials, it was determined that Interferon + Lactobacilli vaginal capsules had the highest probability of being the optimal intervention, as evidenced by its SUCRA value of 0.85 (Fig. 6, Additional file 2: Fig. S5-1 for rankogram). However, it should be noted that this conclusion was limited by the small sample size of only 3 trials and the wide range of 95% CIs. Furthermore, when focusing on the network of high-quality trials, Interferon + Baofukang emerged as the optimal regimen, as evidenced by its SUCRA value of 0.97 (Additional file 2: Fig. S5-2 for SUCRA and Additional file 2: Fig. S6-2 for Rankogram). Additionally, it was observed that the benefits of Interferon + Baofukang were sustained longer than 12 months. In contrast, the study of Interferon + Lactobacilli vaginal capsules was not followed up for longer than 12 months. However, it should be noted that this conclusion was also limited by the small sample size of only 2 trials and the wide range of 95% CIs. Furthermore, it was observed that interventions utilizing a single drug had comparatively lower SUCRA values.

**Table:** 

BFK	BSP	FANIFN	IFN	IFNBFK	IFNLVC	LVC	NT	PTL
0.271	0.533	0.841	0.406	0.795	0.849	0.156	0.013	0.635

Upon conducting a pairwise comparison of high-quality trial networks, it was determined that the joint programs were associated with clearance of excisional treatment postoperative HR-HPV persistent infection (league table appears in Additional file 2: Fig. S6-2), in comparison to single-drug interventions. At the same time there was no difference between the other comparisons. NT was associated with less improvement in the clearance of HR-HPV when administered vaginally compared to the majority of excisional treatments. Additional file 2: Fig. S7-1 displays a league table of all trials considered when pairwise comparisons were made.

## Discussion

Although the success of preventive HPV vaccines, developing antiviral drugs specific to HPV has been met with significant delays. The significance of treatment for individuals currently infected with HPV and SIL cannot be understated. While therapeutic vaccines hold promise, currently, there are no available options as most clinical trials are still in the phase I/II stage, and only a few completed trials have advanced to the next stage [28]. By performing a network meta-analysis of the impact of vaginal administration on HR-HPV clearance after the excisional treatment, this study evaluated the most promising pharmaceutical treatment options currently available.

In this study, we discovered that all kinds of vaginal administration, except the individual use of Lactobacilli vaginal capsules, were more efficacious than no treatment in patients with persistent cervical HR-HPV infection after excisional treatment. Single drug regimes, such as Interferon, Baofukang, Bletilla striata Sanhuang Powder, and Paiteling, which possess properties that alleviate heat and dampness and exhibit both antibacterial and antiviral effects, were individually found to be superior to no treatment in terms of effectively clearing persistent cervical HR-HPV infection after excisional treatment.

In joint drug programs, Interferon + Lactobacilli vaginal capsules emerged as the most effective intervention, significantly outperforming both Interferon and Lactobacilli vaginal capsules alone and being primarily superior to no treatment at all. Interferon + Baofukang emerged as the most effective treatment option in our subgroup analysis after eliminating low-quality literature and only considering RCTs with no less than 12 months follow-up. However, effect estimates of Interferon + Lactobacilli vaginal capsules and Interferon + Baofukang were accompanied by low to very low confidence (AFTER GRADE), which made our recommendation for those two types of vaginal administration much lower. Besides, only 2 RCTs with follow-ups of no less than 24 months were available, making it hard to use them in the network meta-analysis, but this is a more rigorous and clinically significant endpoint. There remains a gap in the guidelines addressing the need for interventions for persistent HR-HPV infection following excisional treatment. HR-HPVs utilize viral helper proteins E5, E6, and E7 to adapt to specific ecological niches, establish a cellular environment conducive to viral replication and persistence, and evade host immune surveillance programs [29]. Several basic experiments have demonstrated that the mechanism of Interferon’s increased susceptibility to growth inhibition by HKc/HPV16 is related to the inhibition of E7 protein expression [30]. Studies by Nawa et al. [31] and Woodworth et al. [32] have reported that IFN-α and IFN-7 inhibited the expression of HPV18 E6 and E7 mRNA in HeLa cells and that IFN-7 transcription reduced E6 and E7 mRNA expression in HPV-immortalized human cervical cells. Interferon as an antiviral therapy is a topic of interest, given that no drugs specifically have been developed to target HPV viruses, as demonstrated in our study. While our data suggest a limited degree of benefit associated with using Interferon alone, the absence of studies with follow-up periods longer than 24 months precludes us from providing evidence of its effectiveness in recurrence and long-term outcomes for patients who have undergone excisional treatment for HSIL. Thus, it cannot yet be recommended as a viable treatment option.

Lactobacilli vaginal capsules, which serve as a form of a bioregulatory agent by supplementing the vaginal environment with exogenous Lactobacillus vaginalis in patients with HR-HPV infection, have been shown to increase the abundance of dominant bacteria in the vagina, ameliorate the unbalanced vaginal microecological environment present in such patients, and enhance the local tissue immunity and self-healing capabilities of the organism. The presence of pathogens such as Chlamydia trachomatis, Trichomonas vaginalis, and herpes simplex virus-2 are associated with HPV-associated cervical neoplastic lesions and cancer. Studies have demonstrated that cervical microbial diversity is diminished, and the cervical microbiome is altered after LEEP [33]. The complexity of the microbiome has been found to play a role in the pathogenicity of HPV infection within the female genital tract. An imbalance characterized by a decrease in lactic acid bacteria and an increase in anaerobic bacteria (e.g., Gardnerella spp., Prevotella spp., and Clostridium spp.) has been associated with an increased risk of persistent HPV infection [34, 35]. The intervention of Interferon + Lactobacilli vaginal capsules provided a more significant benefit than Lactobacilli vaginal capsules alone in this study. However, since the follow-up of this regimen was less than 12 months in all cases, its specific mechanism of action and long-term efficacy still require further confirmation through more rigorous basic and clinical studies. Additionally, this regimen cannot currently be recommended due to a lack of safety studies available.

Moderate to large benefits have been shown with Baofukang alone and with Baofukang in combination with Interferon. Baofukang is a proprietary Chinese medicine composed of curcuma oil and borneol. The potential of Baofukang to be an effective candidate for antibacterial therapy is attributed to its antimicrobial properties and ability to enhance Th1 cellular immunity, Th17 of the innate immune response axis, and vaginal epithelium-derived IgG secretion [36]. While further research is needed to understand its mechanism of action fully, Baofukang may be a viable option for treating HPV infection after HSIL, and its efficacy and safety require further investigation.

### Limitations

This study has several limitations.

1. Only one study had more than 100 participants per group, which may have introduced bias due to small study effects.
2. According to the GRADE framework, the quality of all comparisons was assessed as moderate, low, or very low. 51.6% of the studies had low methodological quality and a high risk of bias. Subgroup analysis excluding these studies resulted in some drugs not being included.
3. Although most of the original studies on the topic have only reported postoperative pathological findings without considering the issue of postoperative incisional margins. Numerous retrospective studies have demonstrated that patients with positive postoperative margins are at a higher risk of experiencing recurrence and progression of CIN [12]. Therefore, it is crucial for future investigators to prioritize the examination of postoperative margins during excisional treatment and to adjust treatment plans accordingly. Differences in the viral load of HR-HPV infection and the extent of CIN lesions in HSIL patients can impact the long-term outcomes of these patients after surgery [11]. However, most of the original studies have failed to stratify the analysis of HSIL patients according to viral load classes or CIN II/III classes, which represents a design flaw in these studies.
4. The SUCRA curve is used to estimate the ranked probability of comparative effectiveness between different therapies, but it has limitations, and the results should be interpreted with caution.
5. The issue of drug safety was not analyzed in this network meta-analysis due to the paucity of reported data on drug safety in the original trials.

## Conclusion

The application of various forms of vaginal administration, except of individual use of Lactobacilli vaginal capsules, is more efficacious than no treatment in patients with persistent cervical HR-HPV infection after excisional treatment. However, all the estimates of the effect size for change in the efficiency of HR-HPV clearance after excisional treatment are uncertain when compared to no treatment due to the long confidence intervals. Besides, we have low confidence in effect estimates and ranking of treatments (AFTER GRADE), due to downgrading for study limitations and imprecision. Therefore, further larger, more rigorous, and longer follow-up RCTs are needed to resolve the uncertainty regarding the efficacy of vaginal administration in reducing viral load and clearing HR-HPV after excisional treatment (Additional file 3).

## Supplementary Information

**Additional file 1.** Detailed search strategy tables for all databases.

**Additional file 2.** Supplementary images for the main text.

**Additional file 3.** GRADE Evidence Rating Scale.

## Data Availability

All data generated or analyzed during this study are included in this published article [and its Additional files].

## Abbreviations

HR-HPV: High-risk human papillomavirus
CC: Cervical cancer
SIL: Squamous intraepithelial lesion
LSIL: Low-grade squamous intraepithelial lesion
HSIL: High-grade squamous intraepithelial lesions
CIN: Cervical intraepithelial neoplasia
RCT: Randomized controlled trail
CKC: Cold knife conization
LEEP: Loop electrosurgical excision procedure
RLU: Relative light units
CO: Cutoff
OR: Odds ratio
CIs: Credible intervals
SUCRA: Surface under the cumulative ranking
