# Supplementary material for: Evaluation of the clinical efficacy of vaginal treatment options for persistent high-risk human papillomavirus infection after excisional treatment of cervical high-grade squamous intraepithelial lesions: a systematic review and Bayesian network meta-analysis
Source: Virol J. 2023 Mar 20;20:47. doi: 10.1186/s12985-023-02001-6 (PMC10026470; doi:10.1186/s12985-023-02001-6)
Supplement: Supplementary file 1 — Additional file 1. Detailed search strategy tables for all databases. [file 12985_2023_2001_MOESM1_ESM.doc]

**PubMed Search History：**

| Search number | Query |
| --- | --- |
| #48 | #24 AND #43 AND #47 |
| #47 | #44 OR #45 OR #46 |
| #46 | placebo[Title/Abstract] |
| #45 | randomized[Title/Abstract] |
| #44 | randomized controlled trial[Publication Type] |
| #43 | #25 OR #26 OR #27 OR #28 OR #29 OR #30 OR #31 OR #32 OR #33 OR #34 OR #35 OR #36 OR #37 OR #38 OR #39 OR #40 OR #41 OR #42 |
| #42 | Vaginal Drug Administrations |
| #41 | Drug Administrations, Vaginal |
| #40 | Administrations, Vaginal Drug |
| #39 | Administration, Vaginal Drug |
| #38 | Drug Administration, Vaginal |
| #37 | Vaginal Drug Administration |
| #36 | Vaginal Instillations |
| #35 | Vaginal Instillation |
| #34 | Instillations, Vaginal |
| #33 | Instillation, Vaginal |
| #32 | Intravaginal Administrations |
| #31 | Administrations, Intravaginal |
| #30 | Intravaginal Administration |
| #29 | Administration, Vaginal |
| #28 | Vaginal Administrations |
| #27 | Administrations, Vaginal |
| #26 | Vaginal Administration |
| #25 | "Administration, Intravaginal"[Mesh] |
| #24 | #15 OR #22 OR #23 |
| #23 | #20 OR #21 |
| #22 | #16 OR #17 OR #18 OR #19 |
| #21 | Electrosurgeries |
| #20 | "Electrosurgery"[Mesh] |
| #19 | conizations |
| #18 | Conisation |
| #17 | Conizations |
| #16 | "Conization"[Mesh] |
| #15 | #1 OR #2 OR #3 OR #4 OR #5 OR #6 OR #7 OR #8 OR #9 OR #10 OR #11 OR #12 OR #13 OR #14 |
| #14 | laser cone biopsy |
| #13 | LEEP |
| #12 | LLETZ |
| #11 | lletz |
| #10 | Cervical Intraepithelial Neoplasia, Grade III/surgery |
| #9 | Intraepithelial Neoplasia, Cervical/surgery |
| #8 | Neoplasms, Cervical Intraepithelial/surgery |
| #7 | Neoplasm, Cervical Intraepithelial/surgery |
| #6 | Intraepithelial Neoplasms, Cervical/surgery |
| #5 | Intraepithelial Neoplasm, Cervical/surgery |
| #4 | Cervical Intraepithelial Neoplasm/surgery |
| #3 | Cervical Intraepithelial Neoplasms/surgery |
| #2 | Neoplasia, Cervical Intraepithelial/surgery |
| #1 | "Squamous Intraepithelial Lesions of the Cervix/surgery"[Mesh] |

**Embase Search History：**

| No. | Query |
| --- | --- |
| #4 | #1 AND #2 AND #3 |
| #3 | 'randomized controlled trial'/exp OR randomized:ti,ab,kw OR placebo:ti,ab,kw |
| #2 | 'intravaginal drug administration'/exp OR 'administration, intravaginal':ti,ab,kw OR 'vaginal administration':ti,ab,kw OR 'administrations, vaginal':ti,ab,kw OR 'vaginal administrations':ti,ab,kw OR 'administration, vaginal':ti,ab,kw OR 'intravaginal administration':ti,ab,kw OR 'administrations, intravaginal':ti,ab,kw OR 'intravaginal administrations':ti,ab,kw OR 'instillation, vaginal':ti,ab,kw OR 'instillations, vaginal':ti,ab,kw OR 'vaginal instillation':ti,ab,kw OR 'vaginal instillations':ti,ab,kw OR 'vaginal drug administration':ti,ab,kw OR 'drug administration, vaginal':ti,ab,kw OR 'administration, vaginal drug':ti,ab,kw OR 'administrations, vaginal drug':ti,ab,kw OR 'drug administrations, vaginal':ti,ab,kw OR 'vaginal drug administrations':ti,ab,kw |
| #1 | 'squamous intraepithelial lesion of the cervix'/exp OR 'neoplasia, cervical intraepithelial':ti,ab,kw OR 'cervical intraepithelial neoplasms':ti,ab,kw OR 'cervical intraepithelial neoplasm':ti,ab,kw OR 'intraepithelial neoplasm, cervical':ti,ab,kw OR 'intraepithelial neoplasms, cervical':ti,ab,kw OR 'neoplasm, cervical intraepithelial':ti,ab,kw OR 'neoplasms, cervical intraepithelial':ti,ab,kw OR 'intraepithelial neoplasia, cervical':ti,ab,kw OR 'cervical intraepithelial neoplasia, grade iii':ti,ab,kw OR 'loop electrosurgical excision'/exp OR 'laser cone biopsy':ti,ab,kw OR 'uterine cervix conization'/exp OR conizations:ti,ab,kw OR conisation:ti,ab,kw OR conisations:ti,ab,kw OR leep:ti,ab,kw OR lletz:ti,ab,kw OR 'electrosurgery'/exp OR electrosurgeries:ti,ab,kw |

**Cochrane Central Search History：**

| No. | Query |
| --- | --- |
| #1 | MeSH descriptor: [Squamous Intraepithelial Lesions of the Cervix] explode all trees |
| #2 | Neoplasia, Cervical Intraepithelial |
| #3 | Cervical Intraepithelial Neoplasms |
| #4 | Cervical Intraepithelial Neoplasm |
| #5 | Intraepithelial Neoplasm, Cervical |
| #6 | Intraepithelial Neoplasms, Cervical |
| #7 | Neoplasm, Cervical Intraepithelial |
| #8 | Neoplasms, Cervical Intraepithelial |
| #9 | Intraepithelial Neoplasia, Cervical |
| #10 | Cervical Intraepithelial Neoplasia, Grade III |
| #11 | LLETZ |
| #12 | LEEP |
| #13 | loop electrosurgical excision |
| #14 | laser cone biopsy |
| #15 | #1 OR #2 OR #3 OR #4 OR #5 OR #6 OR #7 OR #8 OR #9 OR #10 OR #11 OR #12 OR #13 OR #14 |
| #16 | MeSH descriptor: [Conization] explode all trees |
| #17 | Conizations |
| #18 | Conisation |
| #19 | Conisations |
| #20 | #16 OR #17 OR #18 OR #19 |
| #21 | MeSH descriptor: [Electrosurgery] explode all trees |
| #22 | Electrosurgeries |
| #23 | #21 OR #22 |
| #24 | #15 OR #20 OR #23 |
| #25 | MeSH descriptor: [Administration, Intravaginal] explode all trees |
| #26 | Vaginal Administration |
| #27 | Administrations, Vaginal |
| #28 | Vaginal Administrations |
| #29 | Administration, Vaginal |
| #30 | Intravaginal Administration |
| #31 | Administrations, Intravaginal |
| #32 | Intravaginal Administrations |
| #33 | Instillation, Vaginal |
| #34 | Instillations, Vaginal |
| #35 | Vaginal Instillation |
| #36 | Vaginal Instillations |
| #37 | Vaginal Drug Administration |
| #38 | Drug Administration, Vaginal |
| #39 | Administration, Vaginal Drug |
| #40 | Administrations, Vaginal Drug |
| #41 | Drug Administrations, Vaginal |
| #42 | Vaginal Drug Administrations |
| #43 | #25 OR #26 OR #27 OR #28 OR #29 OR #30 OR #31 OR #32 OR #33 OR #34 OR #35 OR #36 OR #37 OR #38 OR #39 OR #40 OR #41 OR #42 |
| #44 | MeSH descriptor: [Randomized Controlled Trial] explode all trees |
| #45 | randomized |
| #46 | placebo |
| #47 | #44 OR #45 OR #46 |
| #48 | #24 AND #43 AND #47 |

**China Knowledge Network database(CNKI) Search History：**

(((((((((((((((subject%='Gong Jing Shang Pi Nei Liu Yang Bing Bian' or title%='Gong Jing Shang Pi Nei Liu Yang Bing Bian') OR (subject%='Gong Jing Ai Qian Bing Bian' or title%='Gong Jing Ai Qian Bing Bian') OR (subject%='Gong Jing Gao Ji Bie Bing Bian' or title%='Gong Jing Gao Ji Bie Bing Bian') OR (subject%='Gong Jing Shang Pi Nei Liu Bian' or title%='Gong Jing Shang Pi Nei Liu Bian') OR (subject%=xls'CIN' or title%=xls'CIN') OR (subject%=xls'HSIL' or title%=xls'HSIL') OR (subject%=’Squamous Intraepithelial Lesions of the Cervix’ or title%='Squamous Intraepithelial Lesions of the Cervix’) OR (subject%='Qie Chu Xing Zhi Liao' or title%='Qie Chu Xing Zhi Liao') OR (subject%=xls'LEEP' or title%=xls'LEEP') OR (subject%=xls'CKC' or title%=xls'CKC') OR (subject%='Huan Xing Dian Qie Shu' or title%='Huan Xing Dian Qie Shu') OR (subject%='Zhui Qie' or title%='Zhui Qie') OR (subject%='Qie Chu' or title%='Qie Chu') OR (subject%=xls'LLETZ' or title%=xls'LLETZ') OR (subject%='Dian Wai Ke Shou Shu' or title%='Dian Wai Ke Shou Shu') AND (old subject='suiji'）OR （topic%=’Randomised Control’ or title%=’Randomised Control’）OR (1 day version of topic=Randomised Control+Randomised Controlled Experiment+Randomised))))

**China Biomedical Literature Service(SinoMed) Search History：**

1# HuanXingDianQieShu OR DianWaiKeShouShu OR QieChuXingZhiLiao OR ZhuiQie

2# LLETZ OR CKC OR LEEP

3# GongJingAiQianBingBian OR GongJingAiQianQiBingBian OR GongJingGaoJiBieBingBian OR GongJingShangPiNeiLiuYangBingBian OR GongJingShangPiLiuYangBingBian OR GongJingShangPiNeiLiuBian

4#Randomised OR Randomised Controlled Trial OR Randomised Controlled OR RCT

5# 1# OR 2#

6# 3# AND 4# AND 5#

**WanFang database Search History：**

((Topic:GongJingShangPiNeiLiuYangBingBian or (Title or keyword:GongJingAiQianBingBian OR GongJingAiQianQiBingBian OR GongJingGaoJiBieBingBian OR GongJingShangPiLiuYangBingBian OR GongJingShangPiNeiLiuBian or CIN or HSIL) and ((Topic:Qiechuxingzhiliao or Title or keyword:ZhuiQie or HuanXingDianQieShu or DianWaiKeShouShu or LEEP or CKC or LLETZ) and (Topic: Randomised Control) or (Title or keyword:Randomised or Randomised Controlled Trial or Randomised Controlled or RCT))
