# Supplementary material for: Evaluation of the clinical efficacy of vaginal treatment options for persistent high-risk human papillomavirus infection after excisional treatment of cervical high-grade squamous intraepithelial lesions: a systematic review and Bayesian network meta-analysis
Source: Virol J. 2023 Mar 20;20:47. doi: 10.1186/s12985-023-02001-6 (PMC10026470; doi:10.1186/s12985-023-02001-6)

**Supplementary Figure 1-1: Network plot of interventions (Excluded Trials at High Risk of Bias)**


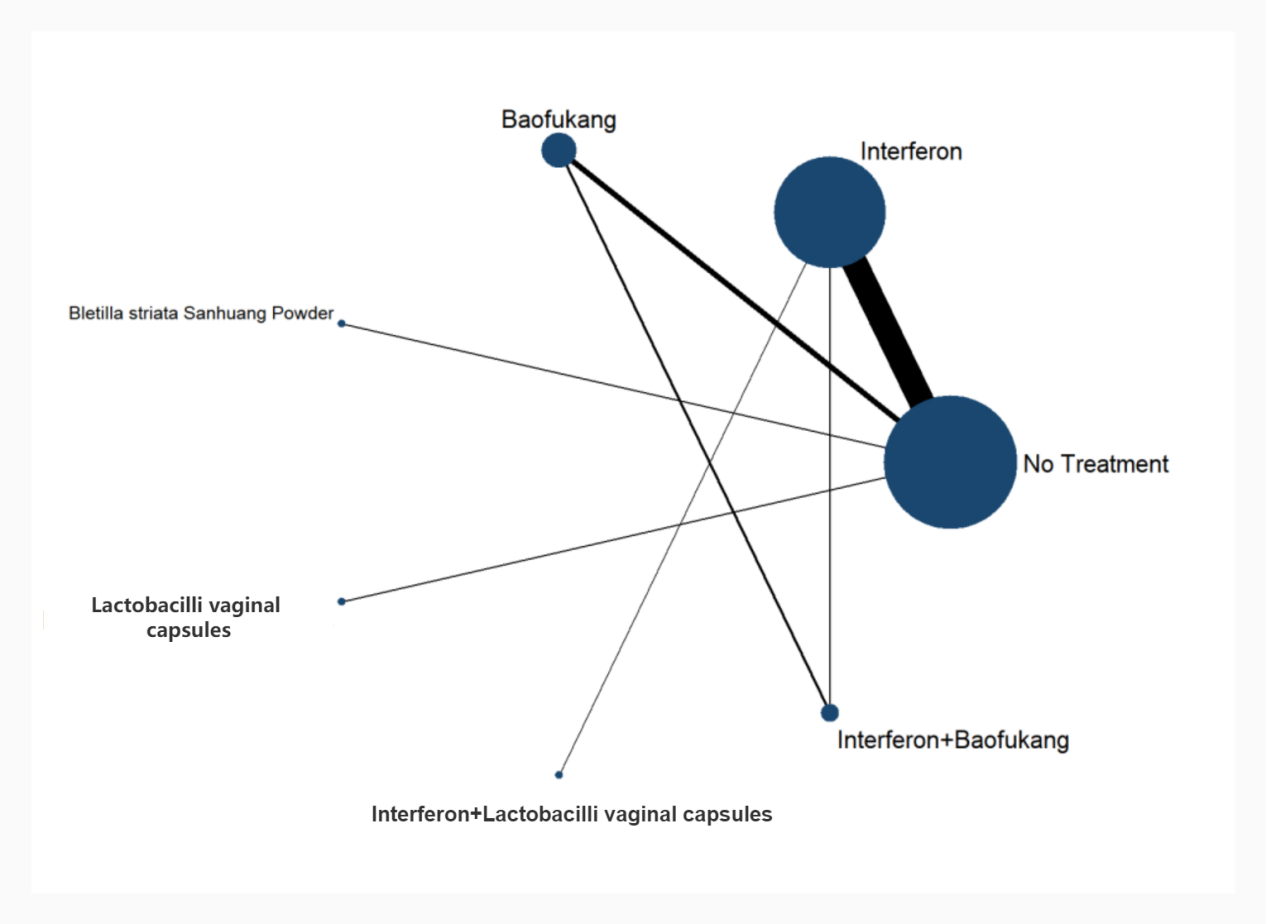


**Supplementary Figure 1-2: Network Plot of Interventions (Excluded Trials that Follow-up Less Than 12 Months)**


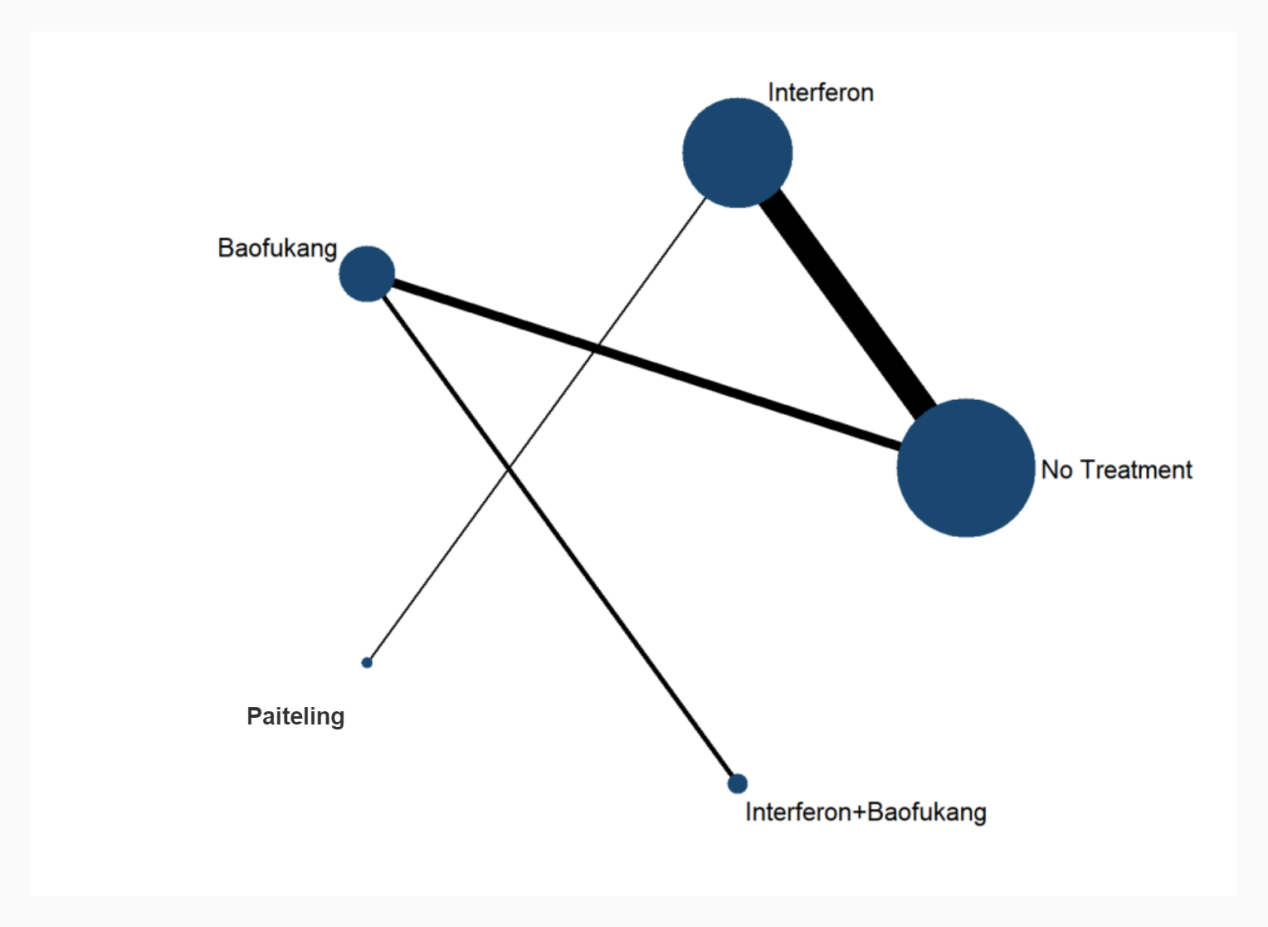


Each circle corresponds to a scenario included in the analysis and its area is proportional to the statistical information (cumulative number of events). Each line represents a direct comparison between interventions and the thickness corresponds to the number of available direct intra-trial comparisons.

**Supplementary Figure 2：Trace and Density Plots**


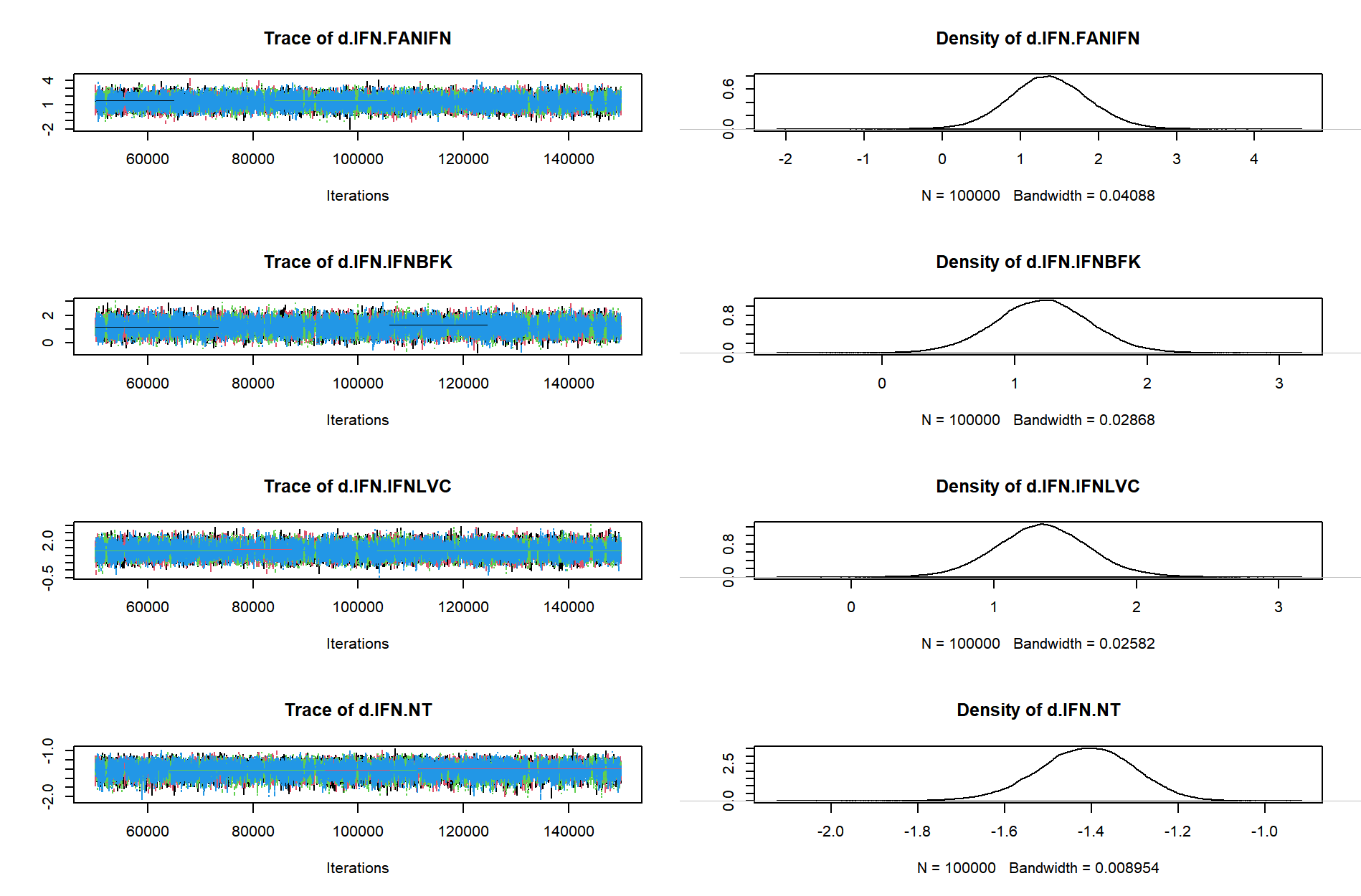


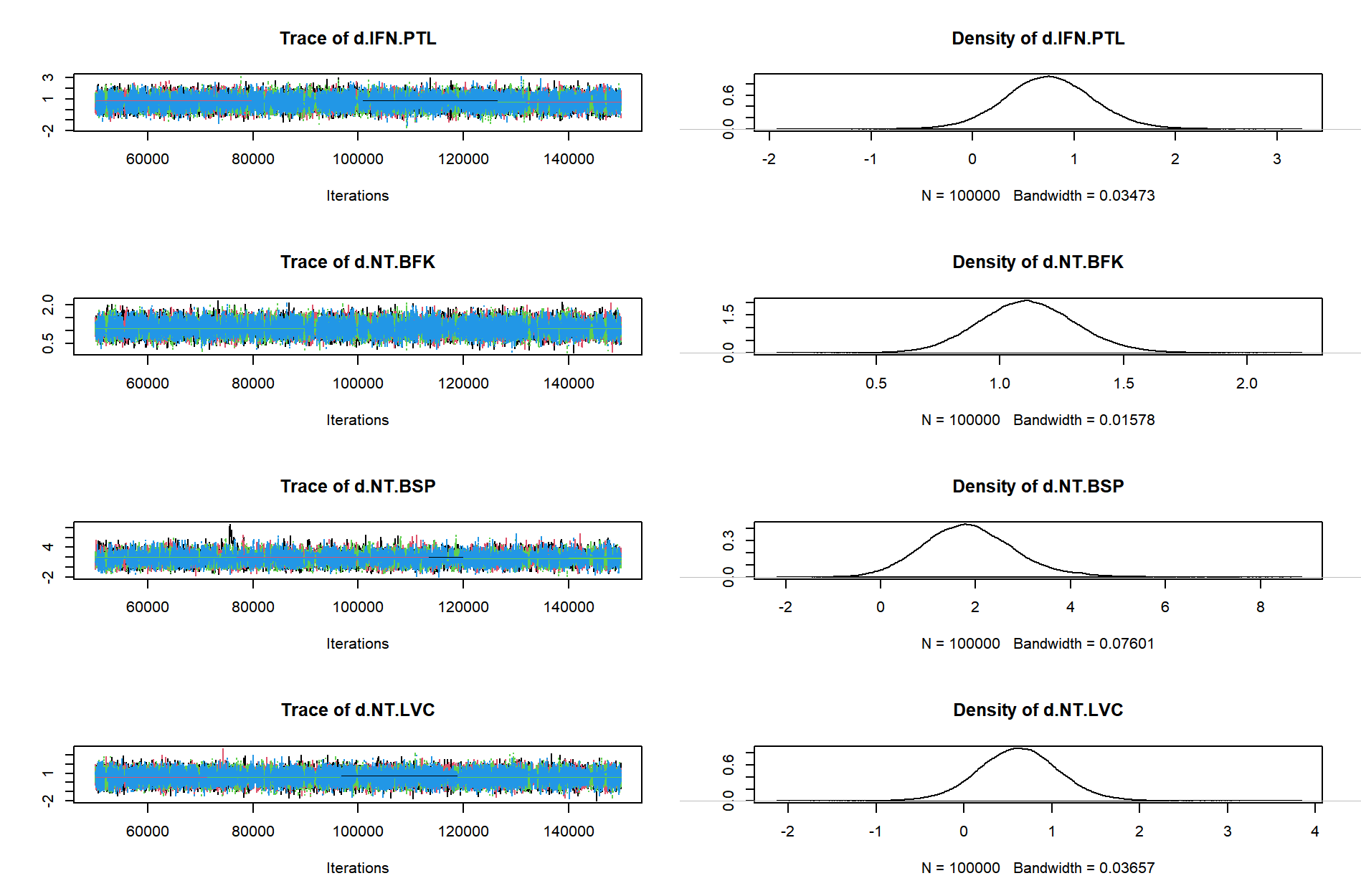


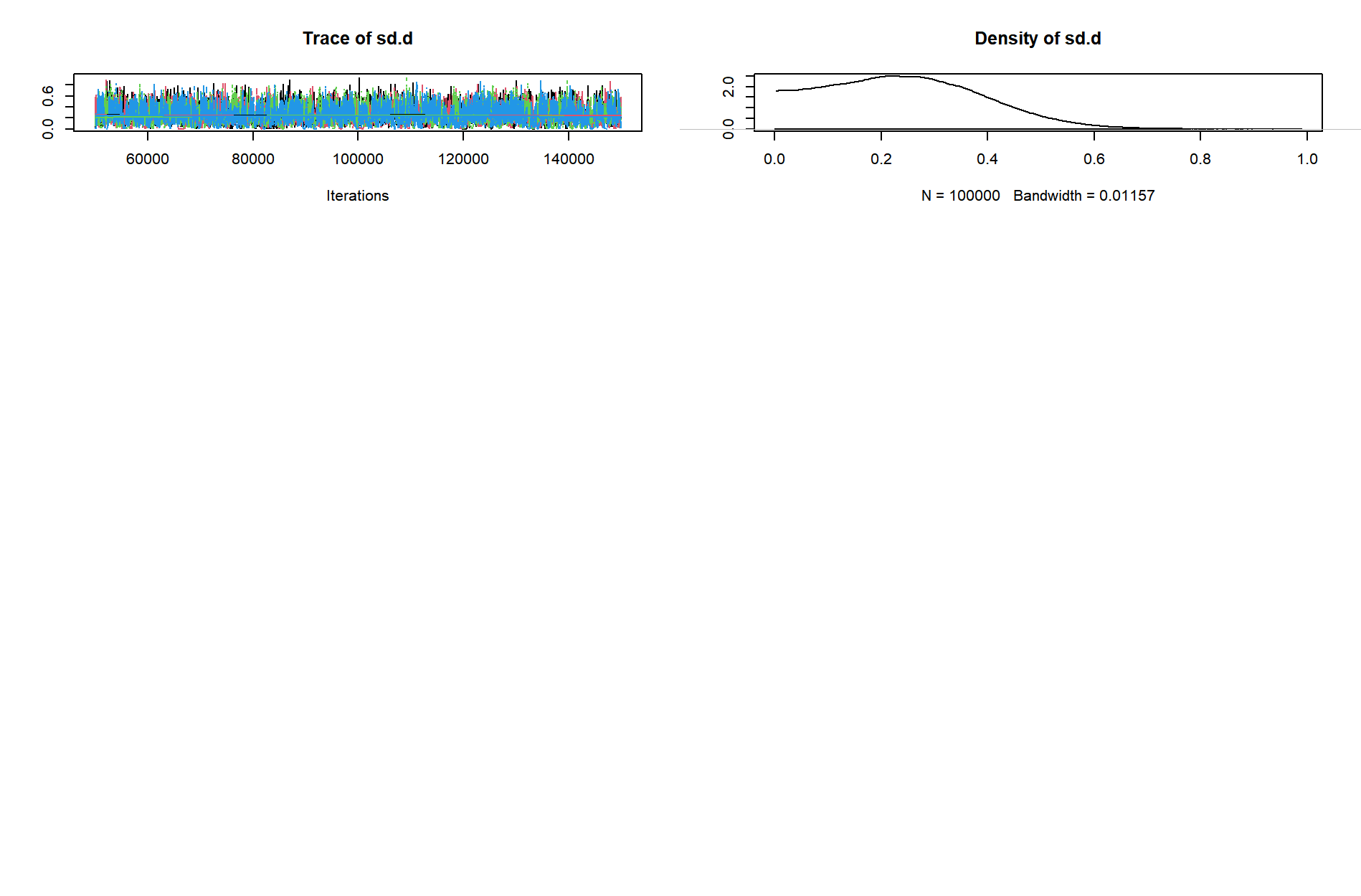


From the trace plot, it can be seen that the MCMC chain fluctuates stably and has good overlap when the number of iterations reaches more than 5000. From the density plot, it can be seen that Bandwith tends to 0 and reaches stability when the number of iterations reaches 20000, which comprehensively indicates that the model converges better.

**Supplementary Figure 3: Brooks-Gelman-Rubin diagnostic chart and PSRF values**


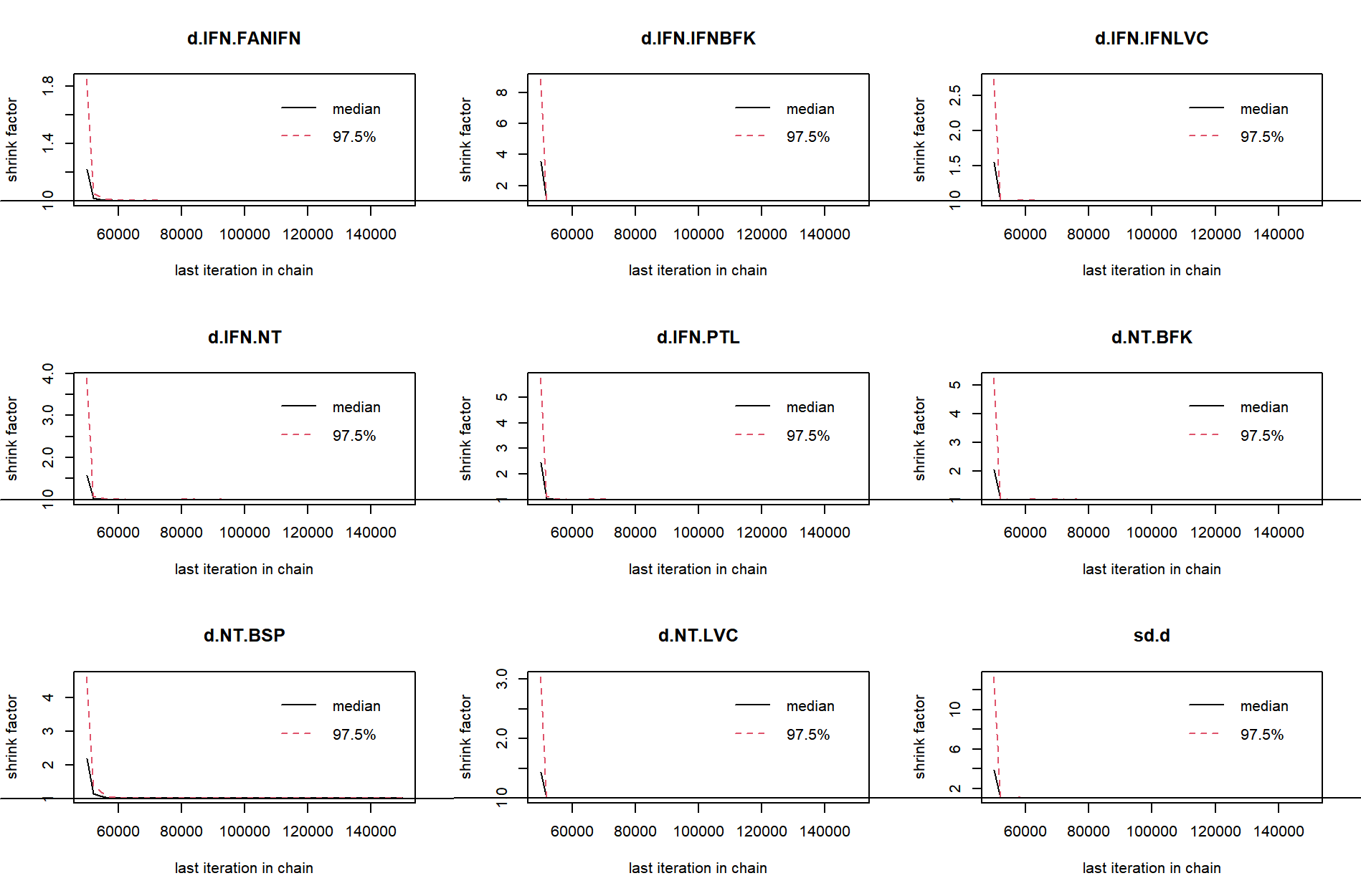


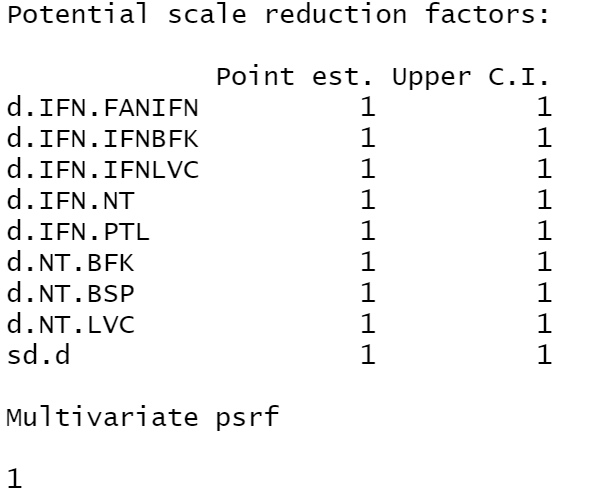


A satisfactory convergence model needs to satisfy three conditions at the same time: (1) the median value of the reduction factor converges to 1 and reaches stability after n iterations of calculation; (2) 97.5% of the reduction factor converges to 1 and reaches stability after n iterations of calculation; (3) the PSRF value converges to 1. As can be seen from the figure, the model satisfies the above convergence is three conditions at the same time, so it is a satisfactory convergence model.

**Supplementary** **Figure 4-1**：**Surface Under the Cumulative Ranking Curve**


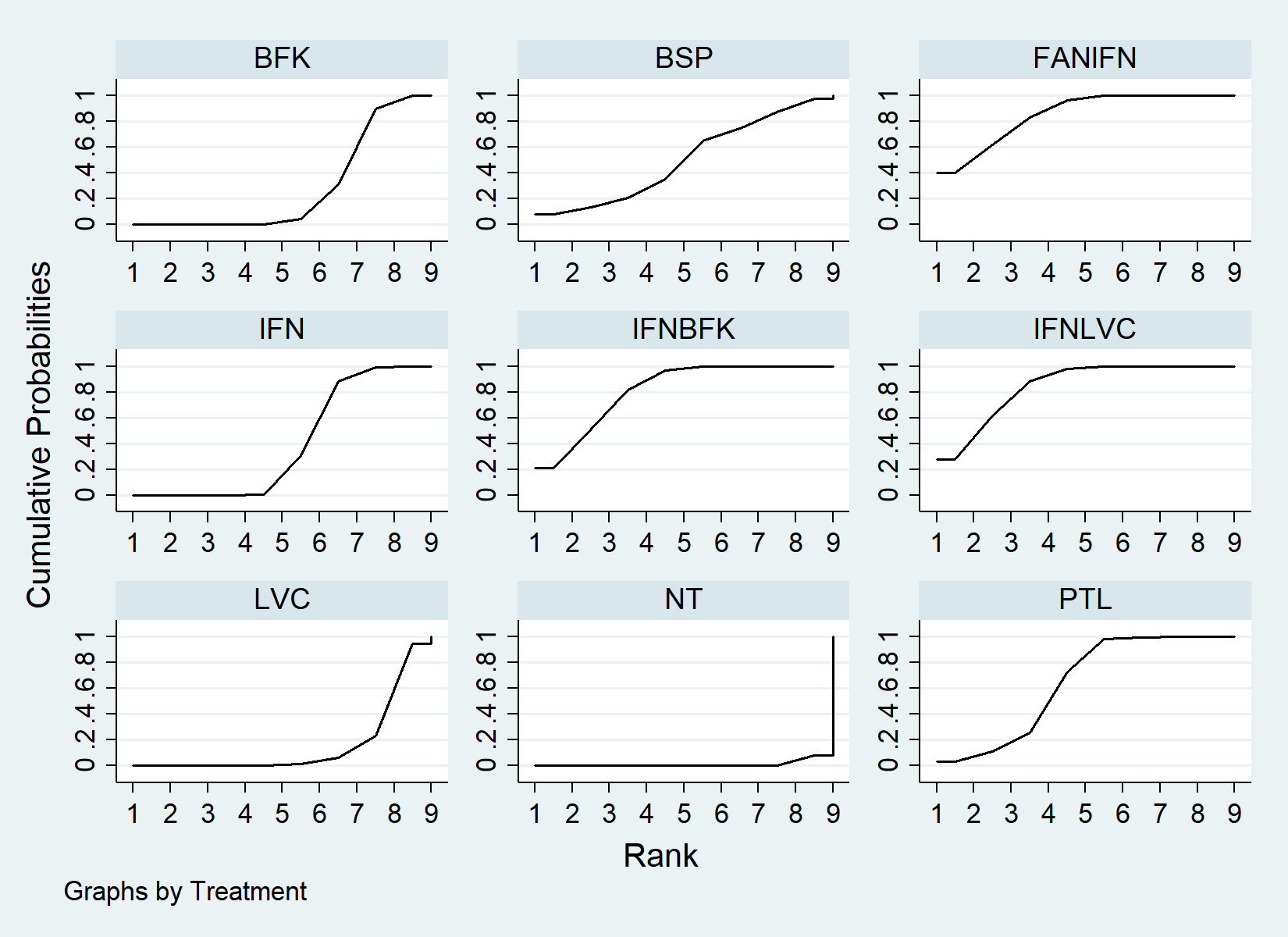


The cumulative ranked area under the curve gives the probability that a treatment will be most effective for HR-HPV clearance after HSIL excisonal treatment. The larger the surface under the curve, the higher the probability of achieving and ranking.

(IFN=Interferon，BFK=Baofukang，PTL=Paiteling，BSP=Bletilla striata Sanhuang Powder，LVC=Lactobacilli vaginal capsules，FAN+IFN=Fuanning+Interferon，IFN+LVC=Interferon+lactobacilli vaginal capsules，IFN+BFK=Interferon+Baofukang, NT=No Treatment)

Rank Probability（preferred direction=1）

BFK BSP FANIFN IFN IFNBFK IFNLVC LVC NT PTL

0.27109125 0.53313219 0.84086562 0.40626781 0.79526719 0.84903312 0.15561656 0.01343625 0.63529000

**Supplementary** **Figure 4-2**：**Surface Under the Cumulative Ranking Curve (Excluded Trials at High Risk of Bias)**


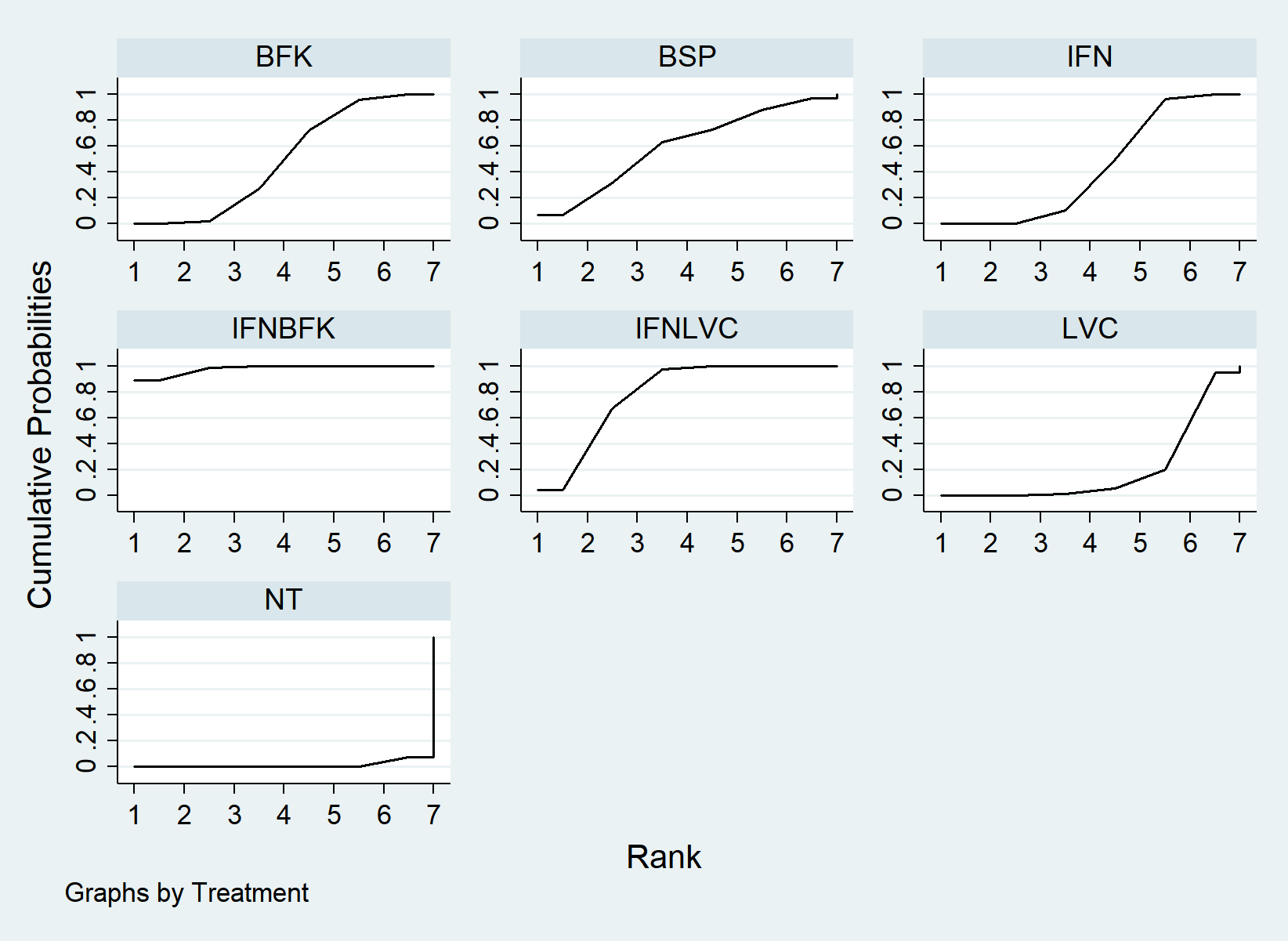


Rank Probability（preferred direction=1）

BFK BSP IFN IFNBFK IFNLVC LVC NT

0.4772983 0.6326987 0.4310954 0.9698683 0.7766150 0.1988717 0.0135525

**Supplementary** **Figure 5-1: Rankogram plot**


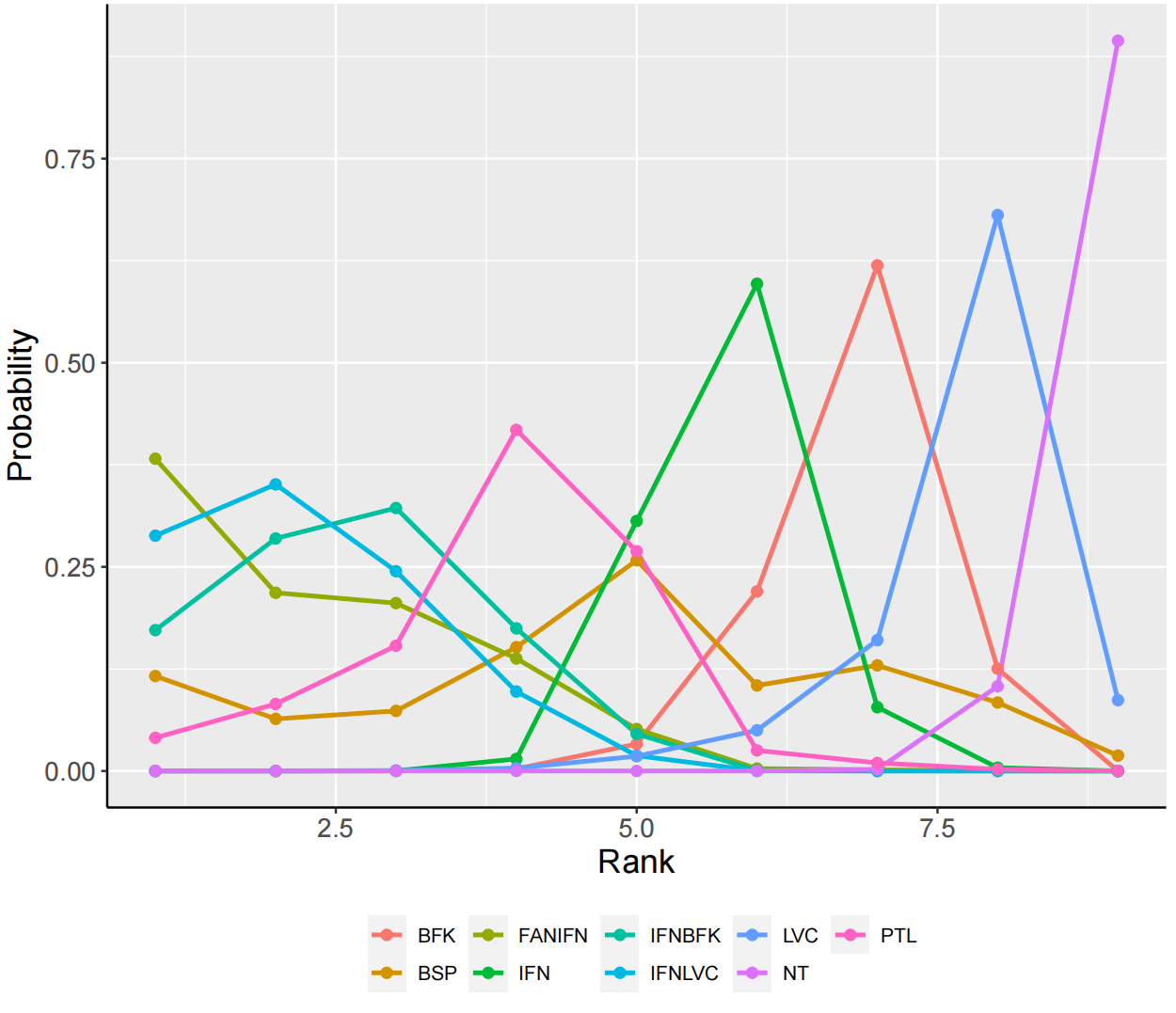


**Supplementary Figure 5-2: Rankogram Plot (Excluded Trials at High Risk of Bias)**


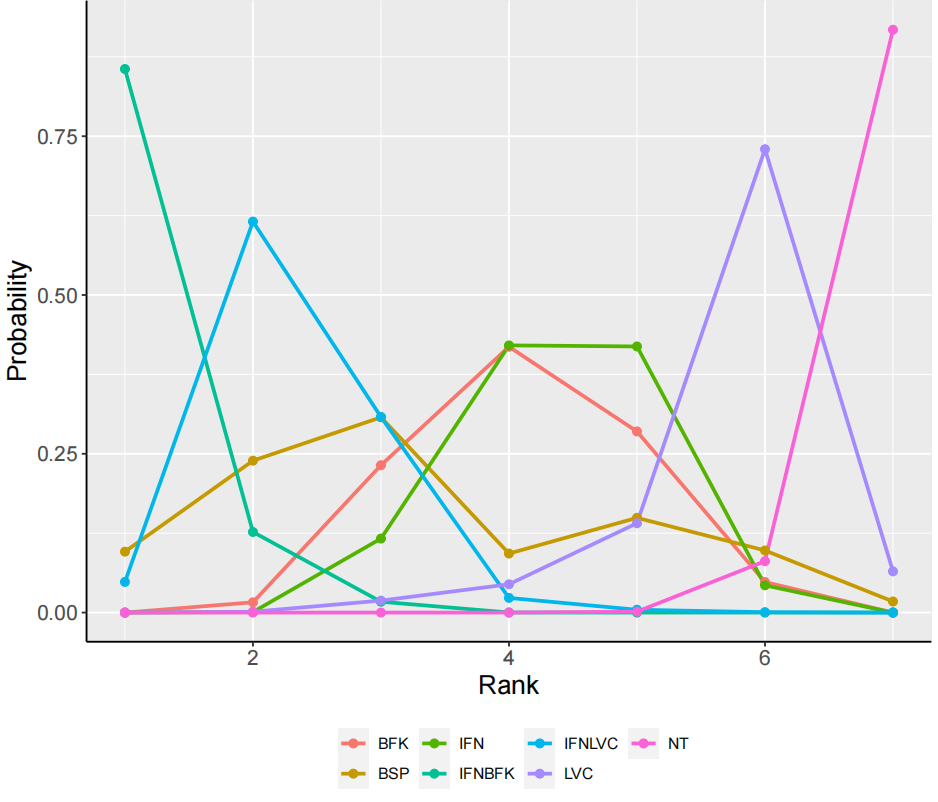


**Supplementary Figure 6-1: league table**

Table legends: Treatment for HR-HPV is reported in relative order of efficacy. Direct comparisons are reported as light gray cells, while significant estimates are shown in bold. Comparisons between treatments read from left to right: network estimates less than 0 indicate that the treatment reported in the column is more effective than the corresponding treatment reported in the row. Point estimates are posterior means.


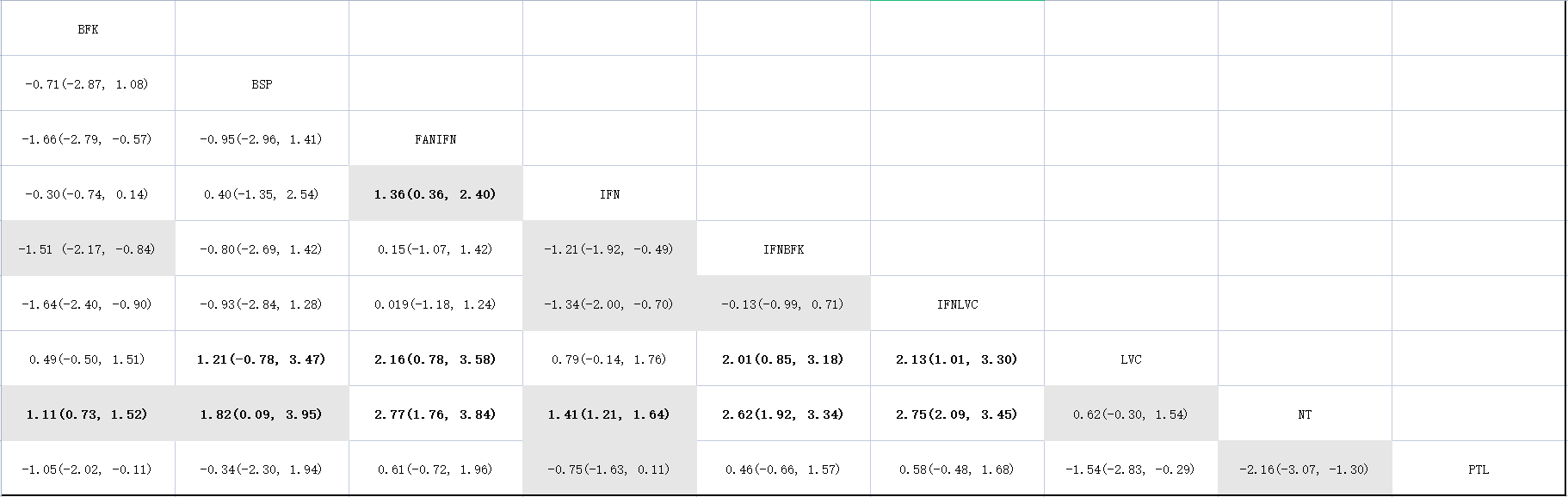


**Supplementary Figure 6-1: league table (Excluded Trials at High Risk of Bias)**


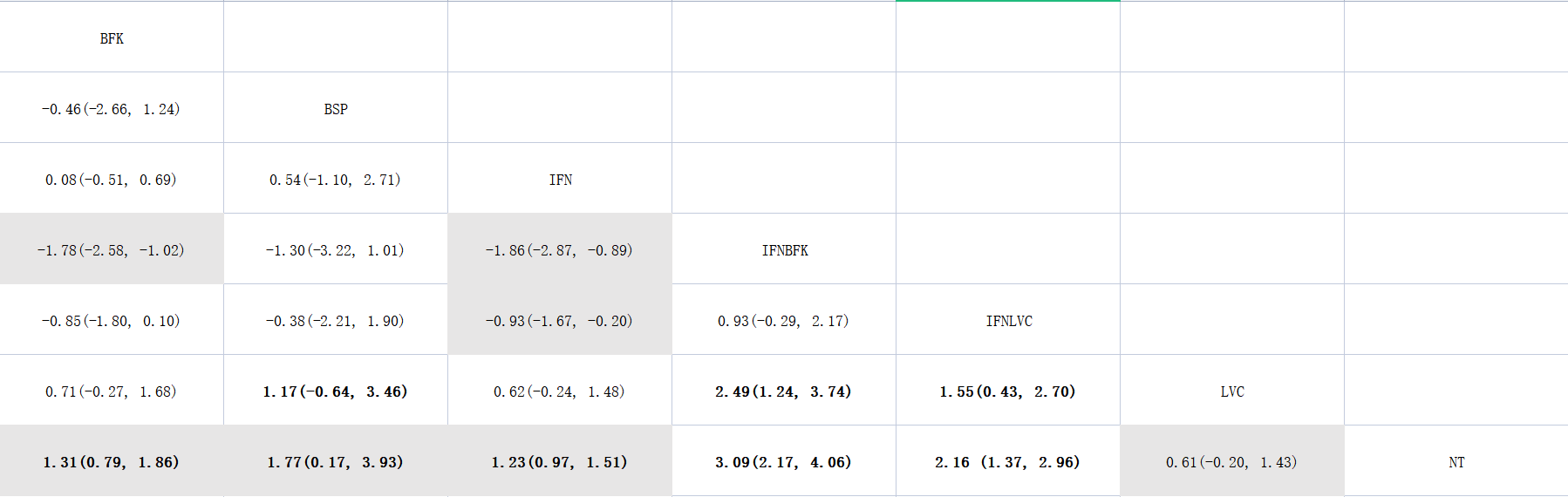


**Supplementary Figure 7：forest plot for pairwise comparison**


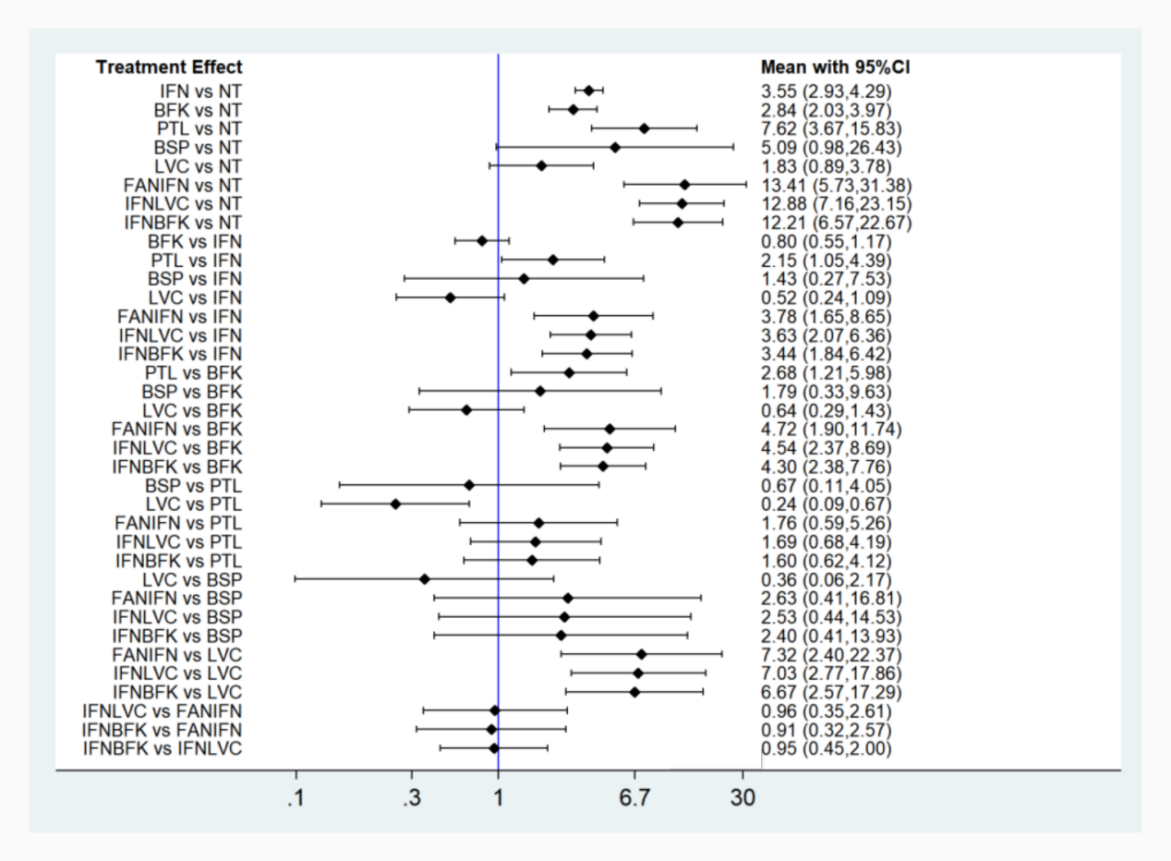

Supplement: Supplementary file 2 — Additional file 2. Supplementary images for the main text. [file 12985_2023_2001_MOESM2_ESM.docx]
